# Recent advances in neuromorphic transistors for artificial perception applications: FOCUS ISSUE REVIEW

**DOI:** 10.1080/14686996.2022.2152290

**Published:** 2022-12-28

**Authors:** Wei Sheng Wang, Li Qiang Zhu

**Affiliations:** a School of Physical Science and Technology, Ningbo University, Ningbo, Zhejiang, People’s Republic of China; b Ningbo Institute of Materials Technology and Engineering, Chinese Academy of Sciences, Ningbo, Zhejiang, People’s Republic of China

**Keywords:** Artificial intelligence, neuromorphic computing, three-terminal neuromorphic transistor, artificial perception systems

## Abstract

Conventional von Neumann architecture is insufficient in establishing artificial intelligence (AI) in terms of energy efficiency, computing in memory and dynamic learning. Delightedly, rapid developments in neuromorphic computing provide a new paradigm to solve this dilemma. Furthermore, neuromorphic devices that can realize synaptic plasticity and neuromorphic function have extraordinary significance for neuromorphic system. A three-terminal neuromorphic transistor is one of the typical representatives. In addition, human body has five senses, including vision, touch, auditory sense, olfactory sense and gustatory sense, providing abundant information for brain. Inspired by the human perception system, developments in artificial perception system will give new vitality to intelligent robots. This review discusses the operation mechanism, function and application of neuromorphic transistors. The latest progresses in artificial perception systems based on neuromorphic transistors are provided. Finally, the opportunities and challenges of artificial perception systems are summarized.

## GRAPHICAL ABSTRACT

**Figure:**
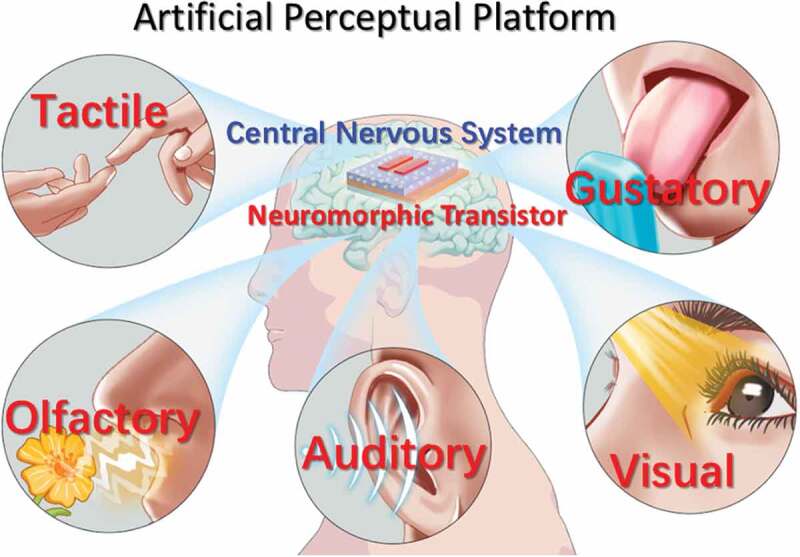

## 1. Introduction

With the rapid developments in information technology and artificial intelligence (AI), the amount of information in today’s society is increasing explosively. Massive data streams need to be processed by computers with excellent performances [1,2] However, such large number of data transmission and storage are still dependent on the conventional von Neumann architecture computing system, which faces the ‘End of Moore’s Law’ and ‘von Neumann bottleneck’ problems, resulting in limited parallel computing and high energy consumption [3,4]. As a comparison, there are ~10^11^ neurons and ~10^15^ synapses within the human brain. It could be deemed as a large-scale parallel, efficient and low energy consumption biological supercomputer with low energy consumption in the order of 10W [5]. Therefore, neuromorphic computing to mimic brain computation mode has been proposed. The concept of ‘neural-inspired’ information processing could be traced back to Alan Turing’s report on ‘Intelligent Machinery’ in 1948 [6]. Until 1990, Carver Mead proposed a new type of electronic hardware for imitating biological information processing in ‘Neuromorphic Electronic System’ [7]. In 1971, Leon O. Chua theoretically proposed the existence of memristor as the fourth fundamental circuit element alongside resistor, inductor and capacitor [8]. It is a two-terminal electronic device with the characteristics of nonlinear resistances, that is, resistance depends on the history of input voltage or current. Thus, the device can demonstrate variable and non-volatile resistances. Until 2008, its existence was proven by the Hewlett-Packard researchers [9]. In a biological neuron, information can be processed and transmitted through electrical and chemical signals in synapse. Thus, synaptic connection strength can increase or decrease after receiving synaptic stimuli. Such an ability is called synaptic plasticity. It plays an important role in the brain. In 1998, a variable conductance device using As_2_S_3_-Ag was proposed to simulate synaptic function [10]. In 2008, Snider, G. S. et al. demonstrated the implementation of spike-timing-dependent plasticity (STDP) learning rule in memristive devices [11]. In 2010, Hasegawa T et al. achieved learning abilities on a single solid-state atomic switch [12]. In 2011, Ohno et al. [13] proposed an Ag_2_S inorganic synapse, which can emulate the synaptic functions of both short-term plasticity (STP) and long-term potentiation (LTP). These pioneering works hint the upcoming of the new era of neuromorphic electronics.

With the developments of new material technology and new conceptual devices, several kinds of neuromorphic devices have been reported to be applied for bionic synaptic functions and neuromorphic engineering, including two-terminal memristors [14–19] and three-terminal transistors [20–23]. Based on these synaptic devices, such as memristors and transistors, several synaptic plasticity behaviors have been implemented successfully, including STP/LTP [24–26], synaptic learning rules [27–29]. Moreover, some advanced neuromorphic functions have also been realized, including pattern classification and recognition [30–33], perception [34–37], logic functions [38–40], conditional reflection [20,41,42], and so on. There are also several review articles on the recent progresses and application of neuromorphic devices [43–45]. Additionally, the development of emerging neuromorphic electronics will also provide extraordinary significance to the realization of artificial perception systems.

**Figure 1.**
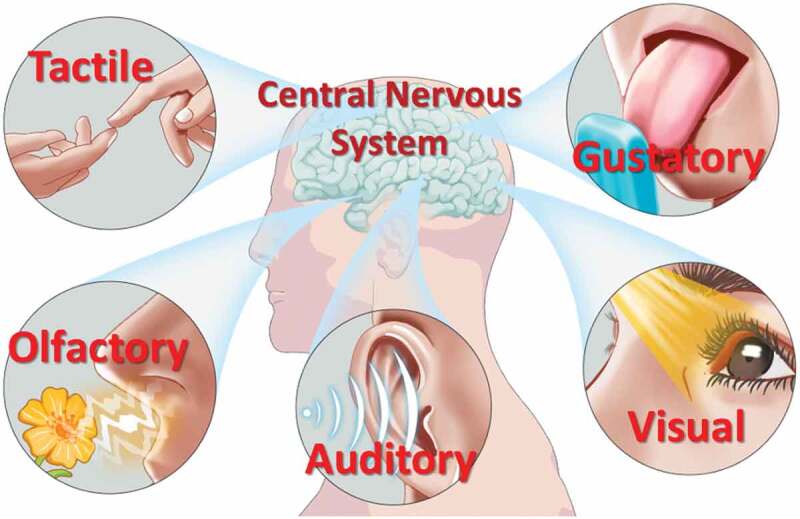
Schematic diagram of human perception system, including visual, olfactory, tactile, gustatory, auditory sense.

In the process of biological evolution, the human body has formed a complex multimodal interactive perception system. Our senses, including vision, touch, auditory sense, olfactory sense and gustatory sense, provide rich information for our brain. Thus, it can interact with the real world vividly and complexly [46,47]. Figure 1 schematically illustrates the human perception systems. Encouragingly, several kinds of materials have been proposed for the fabrication of neuromorphic devices, including two-dimensional materials, optoelectronic materials, organic polymers materials, ionic liquids and ionic gels, ferroelectric materials and biological materials. Thus, various synaptic devices with diverse structures and functions have been greatly developed [42,48–50]. Therefore, neuromorphic devices can not only be used to simulate the basic biological synaptic functions, but also lay the hardware foundation for the construction of artificial perception systems. Up to now, there are many reports that three-terminal neuromorphic devices have been proposed for synaptic bionics and artificial perception [51–54]. However, there are few comments on the latest progresses and a summary of related works. Hence, this review will focus on the research status of neuromorphic transistors in the field of artificial perceptual platform. First, we discuss the working mechanism and biological synaptic function of neuromorphic transistors and introduce the latest progresses of studies in three-terminal transistors. Then, we focus on artificial vision, tactile, auditory, olfactory, gustatory perceptual systems and multi-perceptual fusion systems constructed based on neuromorphic transistors. Next, the existing problems and possible future research directions of the artificial perception systems are discussed. Finally, an outlook is made on the opportunities and challenges of artificial perception systems.

## 2. Three-terminal neuromorphic transistors

**Figure 2.**
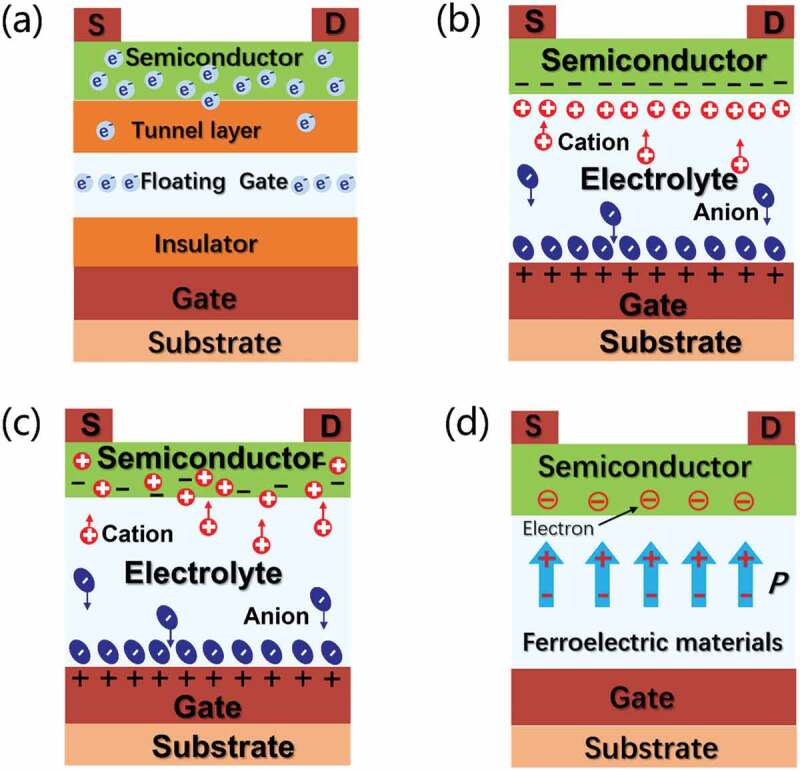
Schematic illustration of (a) Floating-gate transistor (FGT). (b) Electric-double-layer transistor (EDLT). (c) Electrochemical transistor (ECT). (d) Ferroelectric field-effect transistor (FeFET).

Compared with two-terminal memristor-based synaptic devices, three-terminal transistors can perform both program and read operations simultaneously. In other words, information transmission and learning process can execute synchronously. This process is highly similar to the learning process, that is, transmitting neurotransmitters in biological synapses. Therefore, three-terminal transistors-based synaptic devices, that is, synaptic transistors or neuromorphic transistors, have great potentials in the construction of biomimetic synapses and neuromorphic engineering. For bionic synaptic transistor applications, gate electrode and semiconductor channel can be regarded as pre-synapse and post-synapse, respectively. While the variable channel conductance can be regarded as adjustable biological synaptic weights [25]. Up to date, synaptic transistors have been widely proposed to construct neuromorphic platforms and intelligent systems. According to the working mechanisms, three-terminal transistors can be mainly classified into several categories, including floating-gate transistor (FGT) [55,56], electric-double-layer transistor (EDLT) [36,57], electrochemical transistor (ECT) [58,59], ferroelectric field-effect transistor (FeFET) [60,61], and so on. Figure 2 schematically shows different types of transistors.

### 2.1. Floating-gate transistor (FGT)

Figure 2(a) shows a schematic of FGT. It is one of the earliest neuromorphic devices used to simulate synaptic behavior. It possesses similar device structure as conventional FET. The difference is that the control gate in FGT is usually embedded within the dielectric layer [55,62]. When the gate voltage is applied, charges can be trapped in the floating gate layer because of thermal excitation or quantum tunneling. While the charges can be stored in the floating gate layer due to the charge blocking of the tunneling layers. It is worth noting that the floating gate in FGT is electrically isolated, which makes the device exhibit non-volatile changes in the channel conductances. Interestingly, FGT can effectively modulate the trapped charges in the floating gate through gate programming, thus modulating the channel conductances [63]. Thus, FGT can exhibit multi-level conductances, which is conducive to imitating synaptic weight updating.

### 2.2. Electric-double-layer transistor (EDLT)

Figure 2(b) shows a schematic of EDLT. As a kind of electrolyte gated transistors (EGTs), EDLT is a promising candidate for neuromorphic device applications [64–66]. Ionic electrolyte is essentially an electronical insulated but ionic-conducting material. Thus, it (ion conducting materials) can be adopted as gate dielectric in EGTs. The working principle of EDLT is as follows. An EDL is always formed at electrolyte/electrode interface due to the accumulation of ions under external field. Thus, an EDL capacitance above 1.0μF/cm^2^ can be obtained. Because of the strong electrostatic modulation, the EDLT can operate at a low voltage (typically below 3 V). Thus, EDLTs based on different types of electrolytes exhibit ionic/electronic coupling behavior, high carrier concentration and low power consumption [20,67]. Furthermore, thanks to the electrostatic modulation, the EDLTs can produce volatile changes in channel conductances because of unique ion relaxation phenomenon. In other words, the changes of channel conductance is reversible. Such characteristics are proved to be suitable for the simulation of STP. Interestingly, at increased operation voltages, interfacial ionic electrochemical process will occur. In other words, the electrostatic mode will be changed into electrochemical mode, resulting in non-volatile changes in channel conductances, which is suitable for the simulation of LTP.

### 2.3. Electrochemical transistor (ECT)

Figure 2(c) shows a schematic of ECT. ECT is another kind of EGTs. It has also been proved to be promising for neuromorphic engineering applications [68–71]. At low gate voltage, an electric double layer will form at the gate/electrolyte or/and electrolyte/channel interface. However, when the gate voltage exceeds a certain value, ions within the electrolyte will migrate across the interface and will have electrochemical reaction with the channel, which alters the channel conductivities. The process is called electrochemical doping. Similarly, electrochemical de-doping can also occur under reverse voltage. Thus, EGTs under this operation mode could be called as ECTs. Interestingly, the channel conductance could maintain its conductance over a relative long time range under ECT mode, which makes electrochemical transistors be suitable to mimic LTP. In addition, an outstanding aspect of EGTs, including EDLT and ECT, is low power consumption. Due to the strong interfacial ionic/electronic coupling, low power consumption of less than 10f J/spike could be realized, similar to that in a biological synapse [66,68].

### 2.4. Ferroelectric field-effect transistor (FeFET)

Figure 2(d) shows a schematic of FeFET. FeFETs have broad application prospects in artificial synapses due to their easy programming, large switching ratio, low power consumption and non-volatile characteristics. For FeFETs, the ferroelectric dielectric layer is the key to realize the synaptic function. The working principle of FeFETs is as follows. Ferroelectric materials is capable of spontaneous polarization. While the gate voltage will make it reverse in the direction of spontaneous polarization. Furthermore, by controlling the amplitude and time of the gate biases, the polarization inversion can also be obtained accurately, demonstrating non-volatile characteristics [72]. Based on this non-volatile modulation, FeFETs can be adopted to simulate the plasticity of biological synapses [61,73].

In recent years, various dielectrics and channel materials have been used to fabricate synaptic transistors, which not only realize various biological synaptic functions, but also were used to simulate advanced neuromorphic functions. Table 1 summarizes the main synaptic and neuromorphic functions mimicked on three terminal neuromorphic transistors.

**Table 1.** Synaptic and neuromorphic functions mimicked on three terminal neuromorphic transistors.

Type	Channel	Synaptic and neuromorphic functions	Ref
FGT	MoS_2_	STP/LTP, PPF, Pattern recognition	[55] [74] [56] [75] [76]
	Pentacene	EPSC,PPF, PPD,STP,LTP,	
	PDVT-10	EPSC, PPF/D, LTP/D, Pattern recognition	
	Pentacene	EPSC, PPF, Selective Detection	
	CNT	Detect sweet tastants	
EDLT	DNTT	STP, LTP, SVDP	[77] [20] [78] [24]
	ITO	STDP, Pavlovian Associative learning	
	In-Zn-O	EPSC, PPF, STP/LTP, Photoelectric P/D, STDP	
	IGZO	Spatiotemporal integration	
	0D-CsPbBr3-QDs/2D-MoS2	Dynamic filtering, Pavlovian associative learning	[42] [36] [79] [54] [57] [21] [80] [51] [81]
	In_2_O_3_	STP, LTP, 3D-object recognition	
	CNT	Perceptual learning, Pattern recognition	
	ITO	EPSC, PPF, Biological nociceptors,	
	Pentacene	Detect the curvature of the human wrist (0°-90°)	
	IGZO	High-pass filtering, Sound location	
	PTIIG-Np	SNDP, SDDP, SFDP, SVDP, Pattern recognition	
	PDVT-10	Signal recognition, Sound location	
	ITO	Taste aversion learning	
ECT	PEDOT: PSS/PEI	STM to LTM, Pavlovian learning	[82] [58] [83] [59] [84] [85]
	PEDOT: Tos/PTHF	PPF, PTP, STP/LTP, Associative learning	
	PEDOT: PSS	Spatiotemporal dynamics	
	F16CuPc	Distinguish target gases	
	PCDTPT	EPSC, PPF, Simulating gas damage	
	InGdO nanofiber	Gas detection, Conditioning	
FeFET	IGZO	Potentiation, Depression, Pattern recognition	[61] [86] [3] [87]
	IGZO	Pattern recognition	
	AgNWs	PPF, LTP, LTD, Artificial neural network	
	Pentacene	PSC, PPR, Sensory memory	

## 3. Artificial perception system based on neuromorphic transistors

### 3.1. Artificial visual perception system

Human eye is a complex visual system, which receives light stimuli with certain wavelength range from the outside world. The signal can be transferred to cerebral nerve center. Thus, after brain processing and analysis, we can possess visual perception. Studies from neuroscience and cognitive psychology have shown that human visual system is essential for human survival and cognitive behaviors. It is reported that information we received is mainly from visual perception, which is ~80% of the information received by human body [88,89]. Artificial visual perception system inspired by human eyes can realize neuromorphic functions, including recognition, learning, and memorization. As is highly desirable for the next-generation robotics and human-like sensory platforms. Thus, artificial visual perception platform has attracted extensive attention. Recently, artificial synapse devices that can simultaneously process electrical and optical signals have been proposed, demonstrating exciting biological synaptic functions. Besides, neuromorphic visual perception systems based on photosensitive elements and artificial synaptic devices have also been developed. A series of advanced visual perception functions have also been demonstrated. The artificial vision perception systems would be of great significances to the development of neuromorphic electronics in the future.

#### 3.1.1. Optoelectronic synaptic transistors

Among the reported neuromorphic devices, photonic synaptic devices take optical pulse stimuli as input signals. These photonic synaptic devices are expected to be alternatives to the fundamental building blocks of the next generation of high-density, low power consumption and low crosstalk artificial intelligent system [90]. Therefore, photonic synaptic device will play an irreplaceable role in neuromorphic electronics and neuromorphic engineering. In recent years, several kinds of emerging materials have been adopted in photonic synaptic transistors. Moreover, in order to realize the synchronous processing of photoelectric information on neuromorphic devices, photoelectric neuromorphic transistors based on novel transistor architectures have also been developed.

**Figure 3.**
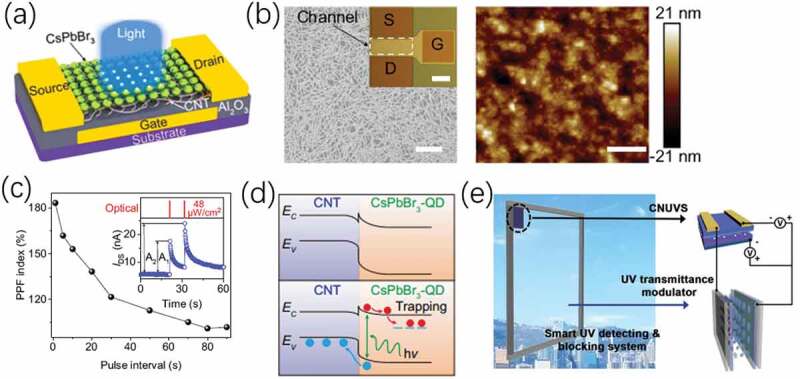
(a) Schematic diagram of phototransistor with a CNT/CsPbBr3-QD channel. (b) Scanning electron microscope (SEM) image of a CNT film and atomic force microscope (AFM) image of a CsPbbr3-QD film. (c) PPF index. (d) Schematic diagram of photogating mechanism. Reproduced with permission [91]. Copyright 2021, springer nature. (e) Smart system to detect and block UV. Reproduced with permission [75]. Copyright 2020, Wiley-VCH GmbH.

Zhu et al. [91] proposed a flexible photoelectric synaptic device, as schematically shown in Figure 3(a). Carbon nanotubes act as active channel. While the all-inorganic perovskite CsPbBr3-QDs quantum dots act as photon active sensing materials. Due to the excellent photoelectric response performances of the CNT/CsPbBr3-QDs structure, several interesting light triggered synaptic responses and synaptic functions were obtained, including paired pulse facilitation (PPF)(shown in Figure 3(c)), the transformation from STP to LTP. The photogating mechanism of the photoelectric synaptic device is schematically shown in Figure 3(d). Due to the energy band mismatch at the CNT/CsPbBr3-QD interface, the built-in electric field forms, equilibrating the Fermi levels. With the light illumination, a highly effective dissociation of photogenerated electron-hole pairs occur at the interface. Thus, negatively charged QDs induce positive carriers in the CNT film through capacitive coupling, increasing the channel currents. Park et al. [75] proposed photonic synaptic transistor adopting carbon nitride (C3N4) as UV-responsive floating-gate layer. C3N4 nanodots dominantly absorb UV light, inducing selective detection of UV light. The synaptic transistor exhibited low energy consumption of only ~18.06 fJ per synaptic event, which is comparable to those of biological synapses. Interestingly, UV responsive synaptic transistors were proposed with an UV-transmittance modulator to develop a smart system to detect and block UV, demonstrating the functions of the retina, as schematically shown in Figure 3(e). This smart system demonstrates potential in advanced electronic skin.

**Figure 4.**
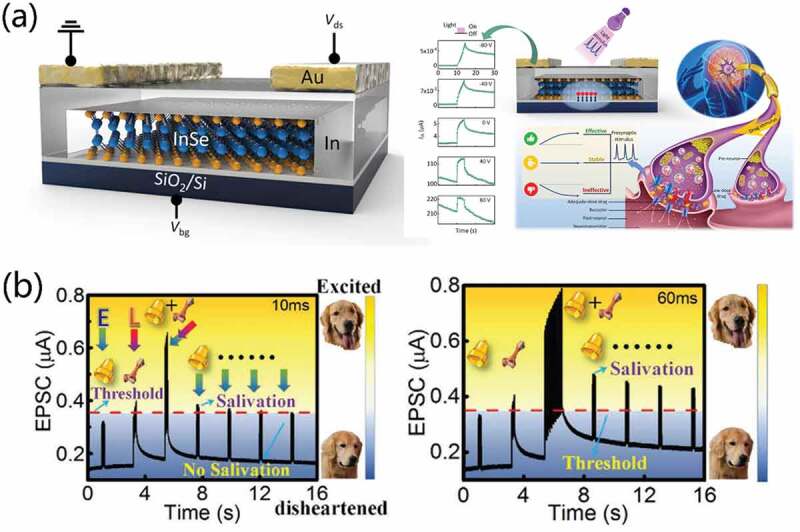
(a) Schematic sketch of the back-gate InSe-based neuromorphic device and schematic diagram of synaptic response mechanism. Reproduced with permission [92]. Copyright 2021, springer nature. (b) Pavlovian conditioning mimicked on vertical van der Waals heterojunction phototransistors based on a colloidal 0D-CsPbbr3-quantum-dots/2d-MoS2 heterojunction channel. Reproduced with permission [42]. Copyright 2020, Wiley-VCH GmbH.

Furthermore, a two-dimensional field-effect transistor based on back-gate structure can adjust channel current, which makes it possible to realize the simulation of synaptic plasticity. With highly gate-modulated and photosensitive of two-dimensional semiconductor material indium selenide (InSe), Nie et al. [92] fabricated a carrier-capture-assisted optoelectronic neuromorphic device based on van der Waals materials with back-gate structure, as shown in Figure 4(a). Owing to the trap capture/release characteristics of InSe/SiO_2_ interface, the highly gate-modulated photoresponse supported the emulation of effective/stable/ineffective feature. The device demonstrate STP, LTP and PPF behaviors. Tuned with photic stimulus, drug-related synaptic plasticity and medicine-acting metaplasticity were emulated.

In physiology, the famous Pavlovian conditioned reflex is the essence of associative learning and memory. Moreover, associative learning and memory play an important role in cognitive behavior and logic thinking. Martin Ziegler et al. [93] demonstrated basic neurobiological phenomena of learning, such as habituation, sensitization non-associative and associative types of learning, on a single memristive device (Pt/Ge_0.3_Se_0.7_/SiO_2_/Cu). Cheng et al. [42] proposed a novel photoelectric neuromorphic device with vertical van der Waals heterojunction phototransistors (MVVHT) based on colloidal 0D-CsPbBr_3_-quantum-dots/2D-MoS_2_ heterojunction channel. Ion gel electrolyte acted as gate dielectric. The device exhibits not only high photoresponsivity but also fundamental synaptic characteristics. Based on 0D-perovskite/2D-MoS_2_ MVVHT, efficiency adjustable photoelectronic Pavlovian conditioning and photoelectronic hybrid neuronal coding behaviors were implemented successfully, as shown in Figure 4(b).

**Figure 5.**
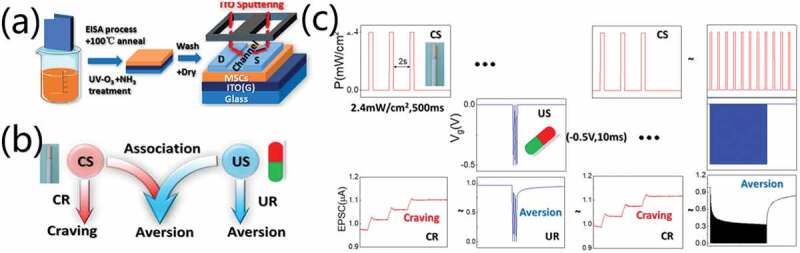
(a) Schematic diagram for fabricating mesoporous silica coating (MSC)-gated oxide photo-perception neuromorphic transistor. (b) Taste aversion learning sketch based on neuromorphic transistor. (c) Realization of taste aversion learning behavior based on photo-perception neuromorphic transistor. Reproduced with permission [81]. Copyright 2020, springer nature.

In biological science, conditioned taste aversion (CTA) or taste aversion learning plays an important role in preventing animals from being invaded by toxic substances. When there are nausea, abdominal pain, diarrhea and other visceral discomfort symptoms with a new sense of smell, animals tend to avoid food with the similar taste characteristics in the future. Thus, CTA has been applied in clinical medicine. The formation of CTA is quite similar to the formation of conditioned reflex. It requires the combination of conditional stimulation (CS) and unconditional stimulation (US). At the beginning, CS can trigger conditioned response (CR), that is, an addictive behavior toward CS. While US can trigger unconditioned response (UR), that is, aversive responses. When applying CS and US simultaneously, associative learning is triggered. Thus, CS can also trigger unconditioned response (UR) when there is no US. Ren et al. [81] proposed a simple aqueous solution-processed mesoporous silica coating gated ITO photo-perception neuromorphic transistor, as schematically shown in Figure 5(a). The neuromorphic transistors exhibit optical and electrical synergic response characteristics. Light stimulation is considered to be CS (cigarettes), while electrical stimulation is considered to be US (drugs). An aversion to learning behavior is successfully simulated on the device with smoking cessation as an example, as shown in Figure 5(b,c).

**Figure 6.**
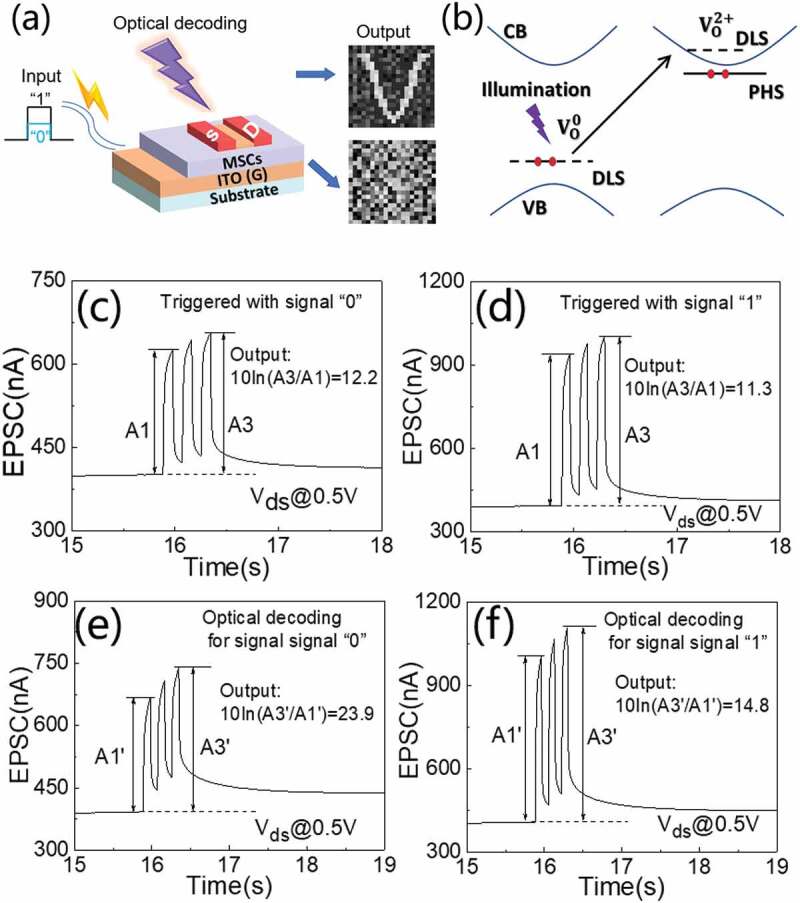
(a) Schematic diagram of the proposed ITO ionotronic neuromorphic transistor with information decoding function. (b) the mechanism for photo-responses. (c) EPSC triggered with signal “0”. (d) EPSC triggered with signal “1”. (e) EPSC with signals “0” decoded with synchronously optical spikes. (f) EPSC with signals “1” decoded with synchronously optical spikes. Reproduced with permission [94]. Copyright 2022, the royal society of chemistry.

Additionally, our brain can process perceptual information with a widely distributed parallel neural network. Thus, it can control advanced neural activities such as conscious, language, learning, memory. Ren et al. [94] proposed a vision enhancement and information decoding platform using aqueous solution-processed mesoporous silica coating gated ITO ionotronic neuromorphic transistor, as schematically shown in Figure 6(a). Excellent electrical performances with the abilities of synergetically responses to both optical and electrical stimuli were demonstrated. The photoresponsive activities were suggested to be originated from the neutral defects in the sputtered ITO, as schematically shown in Figure 6(b). Neutral oxygen vacancies ($V_{O}^{0}$) will bind electrons, resulting in the formation of defect-localized states (DLS) in the forbidden band. Upon photon illumination, $V_{O}^{2+}$ can be generated through photoionization. Then, new DLS and perturbed-host state (PHS) will be generated above or below the bottom of the conduction band (CB), respectively. Thus, electrons will occupy PHS. Such a metastable configuration is conductive because the PHS is very close to the CB, which would result in photo-responses. Thus, they demonstrated information decoding on the ITO photoelectric neuromorphic transistor. They defined 3 successive electrical spikes with an amplitude of 0.1 V as signal ‘0’ and 3 successive electrical spikes with an amplitude of 0.2 V as signal ‘1’, respectively. When triggered with signal ‘0’, the third amplitude of excitatory postsynaptic current (EPSC) (A3) and the first amplitude of EPSC (A1) are ~254 nA and 224 nA, respectively. Thus, an output, defined as 10In(A3/A1), is ~12.2, as shown in Figure 6(c). When triggered with signal ‘1’, the output is ~11.3, as shown in Figure 6(d). Here, the neuromorphic transistor can’t distinguish the signal ‘1’ and the signal ‘0’. As comparison, when illuminated with synchronous optical spikes (2.4 mW/cm^2^, 100 ms), the outputs were modulated to ~23.9 and ~14.8 for signal ‘0’ and ‘1’, respectively, as shown in Figure 6(e,f). The results indicate that the outputs could be distinguished easily. Here, the synchronous photo stimuli would improve the recognition accuracy of input signals. Such optic decoding scheme would have potentials in information processing, machine vision enhancement, and so on.

**Figure 7.**
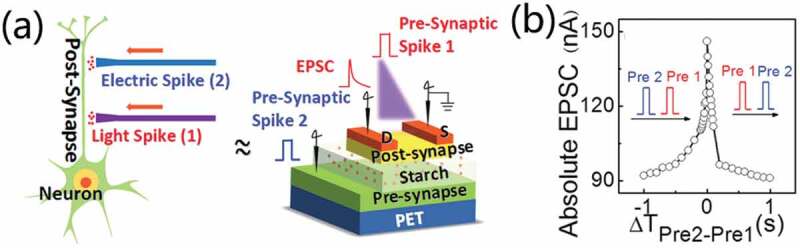
(a) Schematic diagram of photoelectronic synergic coupled IGZO neuromorphic transistor. (b) Absolute EPSC amplitude values at zero time, demonstrating temporal and spatial manner. Reproduced with permission [24]. Copyright 2020, the royal society of chemistry.

Biomaterials are also interesting to fabricate optoelectronic synaptic devices with great potentials in low-cost environment friendly neuromorphic electronics. Guo et al. [24] proposed photoelectronic synergic coupled indium gallium-zinc oxide (IGZO) neuromorphic transistors, as schematically shown in Figure 7(a). Proton conductive starch-based bio-polysaccharide electrolyte acted as gate dielectric. Photoelectric synergic spatiotemporal integration behaviors were demonstrated by introducing light spike stimulus and voltage spike stimulus in a temporal and spatial manner, as shown in Figure 7(b). Furthermore, connections between electric spikes and light spikes have been established, resulting in the demonstration of Pavlovian classical condition.

All these achievements indicate that the proposed photoelectric synergic neuromorphic transistors would have potentials in brain-inspired multifunction intelligent visual perception system applications.

#### 3.1.2. Artificial visual perception

In human visual system, external visual information are recognized by visual perception process in a conjunction with parallel memorizing and learning processes [95]. However, it is a complex process to develop artificial visual perception system that combines retinal perception, information processing and storage. Among various synaptic devices, photoelectric synaptic transistors combine the functions of photosensitivity and information processing. Thus, they are expected to act as candidates for mimicking biological retinal visual perception.

**Figure 8.**
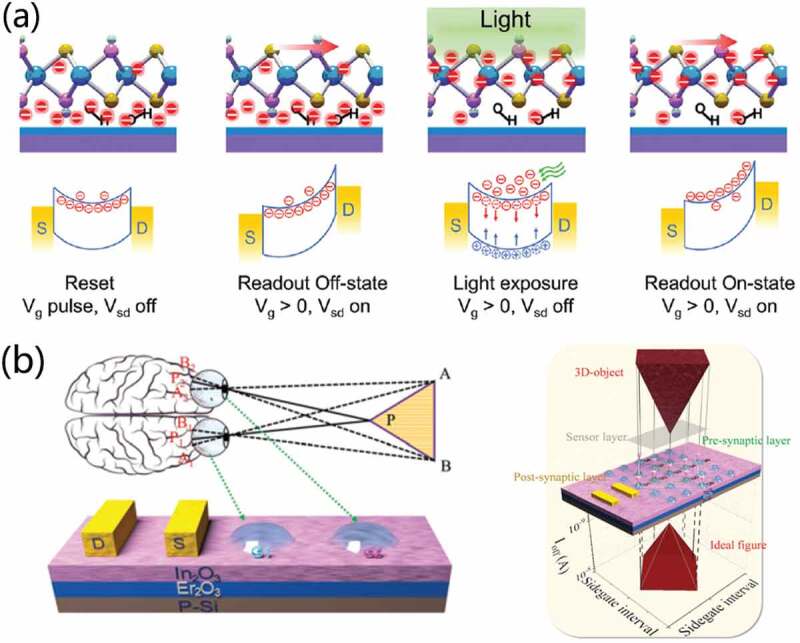
(a) Schematic diagram of the operational mechanism of the MoS2 optical memory transistor. Reproduced with permission [96]. Copyright 2020, the Royal Society of Chemistry. (b) Schematic diagram of the mechanism of binocular vision and the recognition of a 3D cubic cone. Reproduced with permission [36] Copyright 2021, the royal society of chemistry.

Hu et al. [96] developed a novel method to synthesize hydrophilic MoS_2_ monolayers through covalently introducing hydroxyl groups during growth. They propose neuromorphic visual systems consisted of arrays of hydrophilic MoS_2_ monolayer optical memory transistors. Because of strong capability of charge trapping for hydroxyl groups, the hydrophilic MoS2 monolayers can demonstrate excellent electrical, optical, and memory properties. Figure 8(a) shows a schematic of the operational mechanism. The transistor demonstrates excellent light-dependent and time-dependent photoelectric performances. Moreover, it also demonstrates good photo-responsive memory characteristics with multibit storage. The switching ratio is above 10^4^. It is worth noting that the neuromorphic visual system realizes high-quality image sensing and memory with high color resolution. The work provides a new concept to realize image memorization and to simplify pixel matrix preparation process, which is interesting to the development of artificial visual systems. Recently, 3D-object recognition of the stereo vision has also been successfully mimicked by constructing multiterminal neuromorphic transistors. Fan et al. [36] reported PEO and PEO:LiClO_4_ side-liquid-gated In_2_O_3_, pentacene thin-film transistors (TFTs) and 2D-MoS_2_ FETs for synaptic plasticity engineering with proton conducting mechanism and charge trapping engineering. These devices demonstrate both STP and LTP. Interestingly, a proof-of-principle artificial stereo vision system is proposed for 3D-object recognition based on In_2_O_3_/Er_2_O_3_ synaptic transistors (Figure 8(b)), which provides great potential for neuromorphic applications. The work is of interest for constructing spiking neural networks with powerful brain-inspired dynamic spatiotemporal processing. Artificial visual perception system similar to adaptive ambient brightness has also been implemented on side-gate transistors. Jin et al. [95] designed optoelectronic In_2_O_3_ transistor array with negative photoconductivity behavior using a side-gate structure. Screen-printed ion-gel acts as gate insulator. Here, an artificial visual perception system capable of self-adapting to environmental lightness is mimicked using the optoelectronic In_2_O_3_ transistor array. Under different levels of light intensity, self-adaptive behavior of light is demonstrated on the device array. Thus, visual adaption with an adjustable threshold range to the external environment is demonstrated. This work provides a new scheme to environmentally adaptive artificial visual perception system, as is meaningful for future artificial intelligence sensing and neuromorphic electronics.

**Figure 9.**
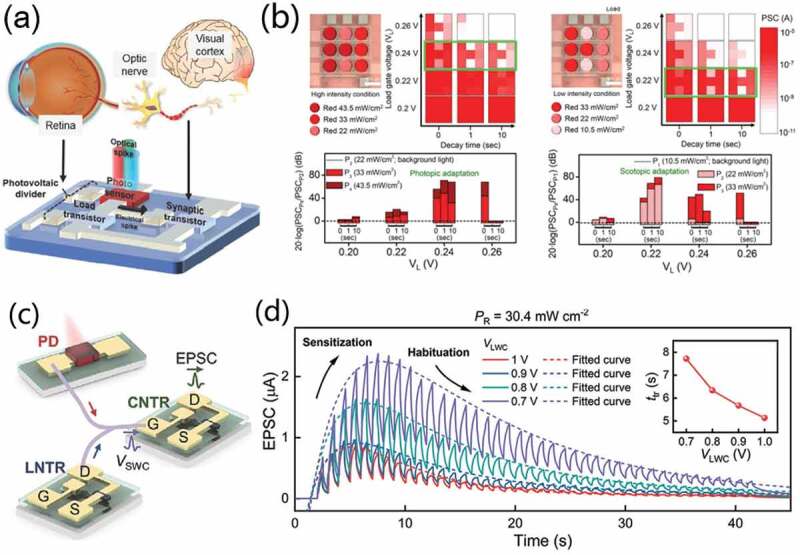
(a) Schematic illustration of artificial optoelectronic neuromorphic device to emulate light-adaptable biological vision function. (b) Environment-adaptable perception behaviors under different light illumination level. (a) and (b) Reproduced with permission [97]. Copyright 2019, WILEY-VCH Verlag GmbH. (c) an integrated DAVAN device. (d) Dynamic EPSC responses of a DAVAN device at different VLWC amplitudes. (c) and (d) Reproduced with permission [98]. Copyright 2021, WILEY-VCH Verlag GmbH.

On the other hand, researchers smartly used photosensitive elements combined with synaptic devices to design various photonic neuromorphic systems. Sung et al. [97] proposed an artificial optoelectronic neuromorphic device array to emulate light-adaptable biological vision function, showing environment adaptive artificial visual perception system (Figure 9(a)), including photopic adaptation and scotopic adaptation. The artificial visual perception circuit includes a photovoltaic divider (CdSe photosensor + a-IGZO load transistor) and a metal oxide ionotronic synaptic transistor (a-IGZO TFT). The photovoltaic divider plays the roles of artificial retina. While the ionotronic synaptic transistor plays the roles of optic nerve. The optoelectronic neuromorphic device demonstrates various visual synaptic functions, including phototriggered STP, LTP and neural facilitation. Moreover, environment-adaptable perception behaviors under different light illumination level were also simulated (Figure 9(b)). Lee et al. [98] demonstrated dynamic artificial visual adaptation neuron (DAVAN) devices based on perovskite photodetectors (PDs) and ion-doped electrolyte neurotransistors, as schematically shown in Figure 9(c). The artificial photoreceptor was consisted of one MAPbI_3_ PD and one (PVPh-Li) solid-electrolyte-based indium-zinc-oxide (IZO) neurotransistors(Li-NTR). Li-NTR, sharing the drain electrode with the MAPbI3 PD, acted as a load neurotransistor (LNTR). A cortical neurotransistor (CNTR) is also adopted, receiving electrical signals through the LNTR and the MAPbI3 PD. The system exhibited strongly changeable adaptability to external stimuli. The MAPbI_3_ PD generated photocurrents in each pixel, and the LNTR adapted to unnecessary information and transmitted the signal to the CNTR. In this artificial visual adaptation neuron (DAVAN) device, the MAPbI_3_ PD acts as the light receiving element of the photoreceptor inside the retina, converting external visual stimuli into electrical signals. This circuit design successfully demonstrated biological sensory adaptation by adjusting the sensitivity of artificial photoreceptors (Figure 9(d)).

#### 3.1.3. Image recognition

Emerging photonic synapses, combining optical and electrical neuromorphic modulation and calculation function, offer favorable choices with high bandwidth, fast speed, low cross-talk and substantially reduced power consumption [99]. Benefiting from the unique and excellent performances, the application of optoelectronic synaptic devices in image recognition has also made great progress in addition to constructing artificial visual perception systems and realizing various advanced neuromorphic functions.

**Figure 10.**
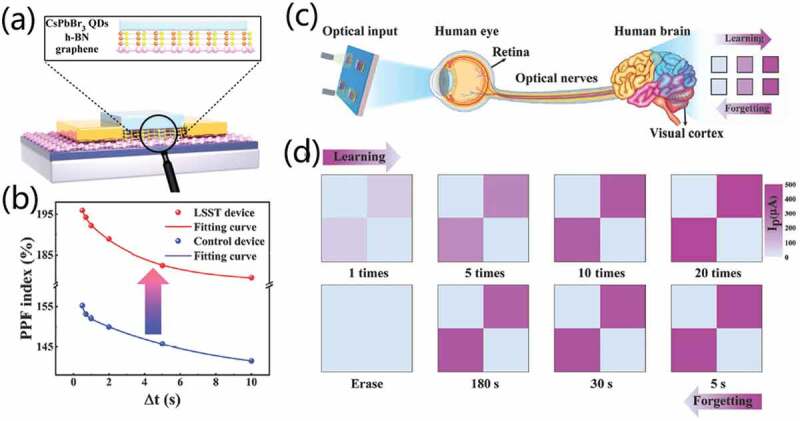
(a) Schematic diagram of LSST device. hBN stands for hexagonal boron nitride. (b) PPF index values. (c) 2×2 pixels imaging chip for imitating visual perception system. (d) Demonstration of dynamic learning and forgetting behaviors. Reproduced with permission [37]. Copyright 2022, WILEY-VCH Verlag GmbH.

Compared with traditional hardware platforms for cognitive tasks, brain-inspired device using analogue weight storage allow to complete cognitive tasks more efficiently. Yao et al. [100] present an analogue non-volatile resistive memory, which shows bidirectional continuous weight modulation behavior. With the device, a neuromorphic network system is constructed for grey-scale face classification. Compared with other hardware platforms, the neural network system has significant energy consumption advantage. Han et al. [37] demonstrated a light-stimulated synaptic transistor (LSST) (Figure 10(a)). It demonstrated an ultra-high PPF index (~196%) based on graphene/h-BN/perovskite QDs triple-layer heterostructure configuration (Figure 10(b)). With light stimuli, the device mimicked typical EPSC behavior of biological synapse. With gate electrical-field, current-reset phenomenon was induced, mimicking inhibitory postsynaptic current (IPSC) behavior of biological synapse. Thanks to the unique optoelectronic characteristics, various types of biological synaptic functions have been simulated, including STP, LTP/LTD, transitions from short-term memory (STM) to long-term memory (LTM), and so on. 2 × 2 pixels imaging chip realizes the functions of real-time sensing and storage functions of visual perception system (Figure 10(c)). The imaging chip also demonstrates dynamic learning and forgetting behaviors, highly similar to that in our brain (Figure 10(d)). Additionally, a two-layer artificial neural network (ANN) was constructed to perform pattern recognition tasks. The recognition accuracy of handwritten digits that adopted from Modified National Institute of Standards and Technology (MNIST) dataset is ~91.5% after 40 epochs.

**Figure 11.**
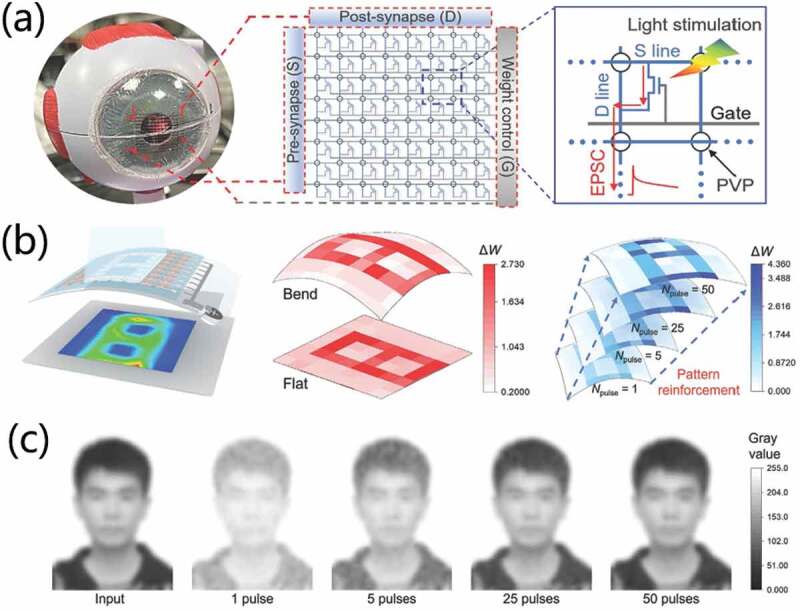
Image recognition and reinforcement learning with FSP-OFETs. (a) Image of the flexible FSP-OFET array attached on human eyeball model. (b) Measured results of a number “8” pattern. (c) Simulated learning results of a human face. Reproduced with permission [22]. Copyright 2022, WILEY-VCH Verlag GmbH.

Besides, Shi et al. [22] reported full-solution printed photosynaptic organic field-effect transistors (FSP-OFETs). Interestingly, a source Schottky barrier is introduced to regulate charge-carrier injection. The FSP-OFET can operate at low working voltage, which greatly reduces the energy consumption. It also provided extraordinary neuromorphic light-perception capabilities. The device demonstrates visual nervous responses to external light stimuli, exhibiting ultralow energy consumption of 0.07–34 fJ per spike in short-term plasticity and 0.41–19.87 fJ per spike in long-term plasticity. As is meaningful for the developments in new-generation visual prosthetics and artificial perception systems. What’s more, an artificial optic-neural network made from an 8 × 8 FSP-OFET array on a flexible substrate shows excellent image recognition and reinforcement abilities at a low energy consumption (Figure 11). As shown in Figure 11(a), the device array can be attached on human eyeball model, which highlights the possibilities of implanting of photosynaptic array into a human eye. It is observed that the reconstructed images can be identified under a flat state and a bending radius of 11 mm. What’s more, the artificial optic-neural network can also exhibit functions of image reinforcement learning, as shown in Figure 11(b). With the reinforcement learning, the evolution of the training process of a human face can be mimicked, as shown in Figure 11(c).

So far, various materials have been adopted to fabricate optoelectronic synaptic transistors [96,101–104], including oxide semiconductor, 2D-layered semiconductor, organic semiconductor, environmentally friendly biomaterials, carbon nanotubes (CNTs), quantum dots (QDs) and ionic gel electrolytes. In addition, various novel structures have also been developed to fabricate optoelectronic devices. A series of biological synaptic functions have also been mimicked, such as EPSC)/IPSC, PPF, STM, LTM, learning-forgetting-relearning activities, and LTP/LTD. Simulator based on ANN is also designed for image recognition [105,106]. The achievements show that optoelectronic synaptic transistors may act as fundamental building blocks in artificial visual perception system.

Though the optoelectronic transistors have excellent photoelectric performances and exhibit basic bionic biological synaptic function, there are still various challenges in neuromorphic computing and complex artificial perception system. On the one hand, most of the proposed optoelectronic transistors operate at single device level, only simulating basic biological synaptic functions. It is an important research branch to integrate high-density phototransistor arrays to realize more complex neuromorphic functions. On the other hand, the proposed optoelectronic synaptic transistors are relatively large, which is not conducive to the integration of optoelectronic synaptic devices. Adopting nanofabrication processing technology to reduce device size while reducing the energy consumption of optoelectronic synaptic devices and obtaining high consistency and stability of optoelectronic synaptic devices is also an indispensable step for the future developments.

### 3.2. Artificial tactile perception system

Tactile sensation is one of the five sense systems of human beings. It can transform external information into inner feelings, which is the earliest developed, the widest distributed, and the most complicated sensory system. Physiologically, touch is detected by receptors on sensory neurons embedded in the skin. The tactile receptors in the skin can convert the external touch/press stimulus into electrical signals, which induce firings of postsynaptic spikes by neurotransmitters [54,67]. These spikes are then transmitted to the terminal somatosensory cortex to form tactile sensation. In addition, tactile perception relies on comprehensive activities of sensing, refining, and learning, which enormously shapes our interactions with external environment. Therefore, developments of artificial tactile sensing system is crucial for the application of robots and prosthetics. In recent years, the integration of different tactile sensors and neuromorphic devices to build artificial tactile sensing system has been reported. Various tactile sensors based on different conduction mechanisms have been explored, including resistive-type, capacitive-type, piezoelectric-type and triboelectric-type tactile sensors.

#### 3.2.1. Artificial tactile system based on resistive-type tactile sensor

**Figure 12.**
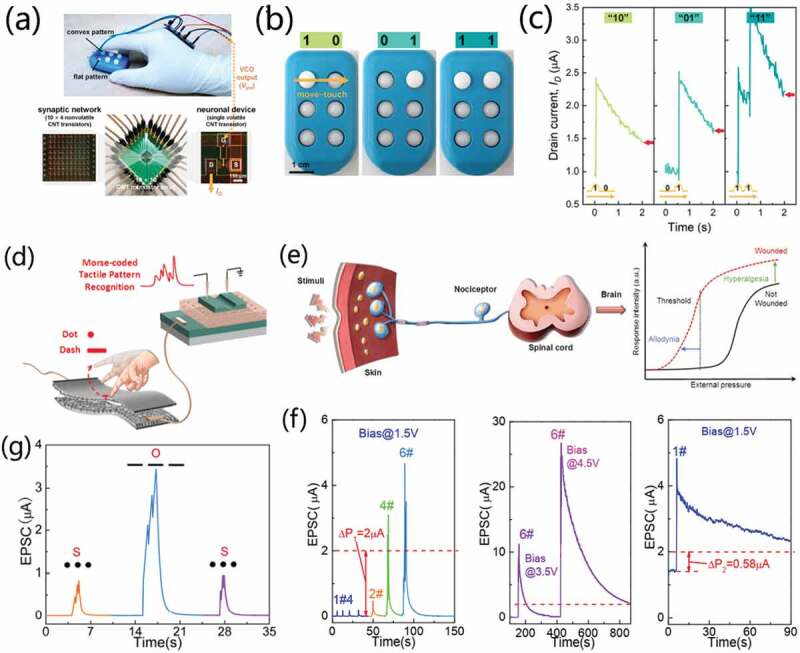
(a) Photograph of the entire tactile sensor system mounted on a hand. (b) Photographs showing the pattern pairs. (c) Typical ID responses for three types of pattern pairs. Reproduced with permission [79]. Copyright 2020, springer nature. (d) Schematic diagram of bionic tactile afferent nerve based on neuromorphic transistors. (d) Schematic diagram of the human nociceptive afferent nerve. (f) Nociceptive abilities emulated on the artificial tactile perceptual neuron. (f) Morse codes data processing. Reproduced with permission [54]. Copyright 2020, American chemical society.

As shown in Figure 12(a), Kim et al. [79] demonstrated a biorealistic tactile sensor system. The artificial tactile sensing system is composed of tactile sensors, voltage-controlled oscillator (VCO) circuits, neuron carbon nanotube (CNT) transistors and CNT synaptic transistor arrays, acting as the sensing receptors, action potentials, neurons and synaptic networks in biological system, respectively. Interestingly, the semivolatile transistor can switch the operation mode based on the bias conditions. Thus, a single device type is allowed to play two different roles (neuronal and synaptic functions) simultaneously. The tactile sensor converts pressure stimuli into resistance changes. In addition, the tactile sensor system can distinguish temporally correlated pressure stimuli, so as to realize pattern recognition (Figure 12(b,c)). In a 10 × 10 CNT transistor array, one CNT transistor acted as neuronal device that operated in volatile mode. Other 10 × 4 CNT transistors operated in a nonvolatile mode acted as a synaptic network to classify the pattern of input pressure. Besides, the learning and recognition process of biological real perception is demonstrated. It is worth noting that the recognition accuracy can be improved through an iterative learning process, which is highly similar to the biological perception learning process.

Yu et al. [54] rationally designed and realized a simple, direct, and inexpensive approach to create artificial tactile perceptual neuron composed of a resistive E-skin device and a chitosan-gated oxide neuromorphic transistor, as schematically shown in Figure 12(d). The tactile sensor is fabricated with solution processing by using KOH solution etched textured silicon wafer as mold. Anisotropic wet-chemical etching of crystalline Si in alkaline solution leads to the pyramidal surface topography with typical height of ~2 μm. The PDMS solution was casted onto alkaline textured crystalline Si surface. After solidification, PDMS membrane with reversed random pyramid patterns can be obtained. Thus, flexible microstructure resistive tactile sensor can be obtained by placing two Ag/ITO/PDMS membranes in a face to face mode. Such microstructured polydimethylsiloxane (PDMS) based resistive E-skins demonstrated a wide detectible pressure range. The sensitivities are estimated to be ~ 2 kPa^−1^ and 0.008 kPa^−1^ in the range of 0 ~ 10 kPa and 10 ~ 100 kPa, respectively. The chitosan-gated oxide neuromorphic transistor demonstrate good electric performances. The current on/off ratio, subthreshold swing, threshold voltage and field-effect mobility were estimated to be ~ 1.3 × 10^8^, ~67 mV/dec, ~-0.02 V and ~23 cm^2^/Vs, respectively. The device also demonstrate good stabilities against bending stress. The chitosan-gated ITO neuromorphic transistor was integrated with the proposed E-skin to construct artificial tactile perceptual afferent nerve. Typical synaptic functions could be triggered with pressure stimuli, including EPSC and PPF. Moreover, pressure amplitude-dependent and pressure duration-dependent EPSCs were demonstrated. At a loading pressure of 1.1 kPa, the peak EPSC is only ~1.2 nA, corresponding to energy consumption of ~0.7 nJ. Interestingly, the artificial tactile perceptual neuron demonstrate a low energy consumption of ~0.7 nJ at a low loading pressure of 1.1kpa. The artificial tactile perceptual neuron can also emulate the nociceptor in biological nerve system, including relaxation, threshold, allodynia, and hyperalgesia, as shown in Figure 12(e,f). Pain threshold (P_th_) is set at a synaptic weight of 2 μA. When applying noxious stimuli (7.84 kPa, 0.1 s) on E-skin, the peak EPSC value is below P_th_. With multi noxious pressure stimuli, the artificial tactile perceptual neuron begins to perceive transient pain. When the E-skin is biased at 3.0 V and 4.5 V, long-term potentiated channel conductance will be triggered. Under the pressure stimuli, allodynia characteristic of the nociceptor can be mimicked. Now, a small noxious pressure will also induce a high EPSC above P_th_, resembling the hyperalgesia characteristic of the nociceptor. More interestingly, the artificial tactile perceptual neuron can also decode Morse-coded external pressure information (Figure 12(g)).

**Figure 13.**
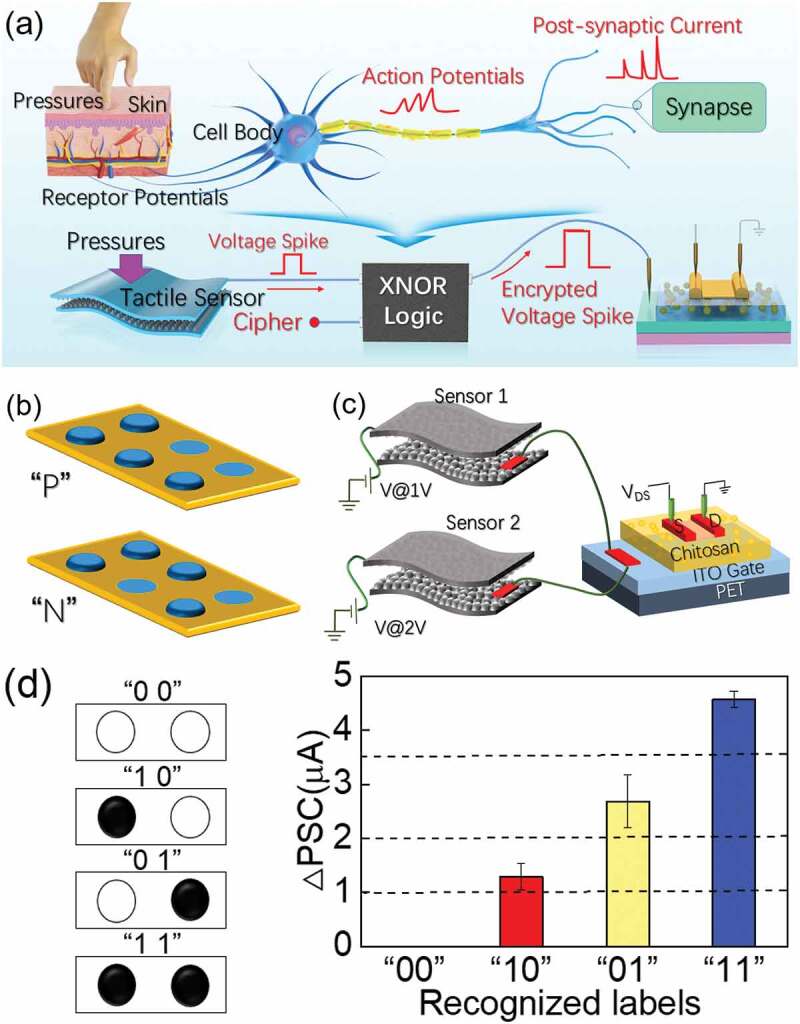
(a) Schematic diagram of simplified biological cutaneous afferent nerves and the flexible tactile perception platform for information encryption applications. (b) Schematic diagram of two Braille codes of “P” and “N” and (c) a single tactile perceptual interactive platform connected with two tactile sensors. (d) Absolute EPSC values when touching patterns of “00”, “10”, “01”, “11”. Reproduced with permission [105,107]. Copyright 2021/2022, IOP.

Generally, artificial perception system need information-receiving unit (that is, the sensory unit) that can obtain real-time information from external environment and information processing unit that can deal with information efficiently. However, information sensed by sensors can be easily obtained illegally during transmission in artificial perception system. Therefore, it is of great interests that information encryption strategy can be developed to ensure the security of information transmission. Shi et al. [107] proposed a flexible tactile perception platform for information encoding and encryption applications, as shown in Figure 13(a). To encrypt the tactile perceptual signals, an XNOR logic circuit was adopted. Thus, information transmitted in the whole perception platform is the encrypted information, avoiding the illegal acquisition of tactile signals. At the same time, the signals can also be decrypted with XNOR logic circuits. Braille is a special text designed for the blind and can be recognized by tactile perception. Each Braille code consists of three rows of patterns. Each row of pattern contains two dots (convex dot or flat dot). Thus, different letters can be represented by the combination of convex dots, as schematically shown in Figure 13(b). Chai et al. [105] also adopted high-sensitive flexible tactile perceptual interactive platform by integrating PDMS-based flexible tactile sensors and a flexible chitosan-gated oxide neuromorphic transistor. The flexible tactile perceptual platform also demonstrate high sensitivity. When loading a low pressure of ~1.4 Pa, the flexible tactile perceptual platform demonstrates high S/N value and sensitivity of ~4.93 and ~6.9 dB, respectively. They demonstrate Braille code recognition functions by integrating two tactile sensors, as schematically shown in Figure 13(c). Tactile sensor 1 and tactile sensor 2 were deemed as fingers that could recognize Braille codes. When the finger touched the convex dot, an EPSC current will be triggered, that is, ‘1’ state. When the finger has touched the flat dot, no EPSC current will be triggered, that is, ‘0’ state. Thus, four states can be defined, including ‘00’, ‘01’, ‘10’ and ‘11’, as shown in Figure 13(d). Therefore, the Braille code can be recognized line by line.

#### 3.2.2. Artificial tactile system based on capacitive-type tactile sensor

In addition to the above-mentioned resistive tactile sensors, capacitive tactile sensors based on organic transistors have also been proposed. Taking the capacitive-type organic transistor as an example, its dielectric layer is similar to the parallel plate capacitor. The pressure stimulus exerted on a transistor could lead to the deformation of dielectric layer, resulting in the changes of capacitance. Therefore, such capacitive-type organic transistor-based tactile sensors can detect the tactile pressure signal via the modulation of capacitances of dielectric layer in a transistor.

**Figure 14.**
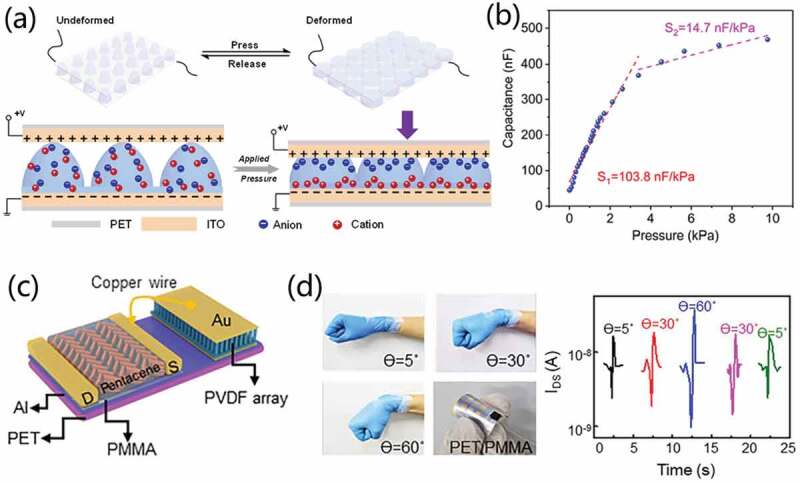
(a) Schematic diagram of the micropatterned EIPH-based capacitive pressure sensor. (b) Capacitance change with respect to the applied pressure. Reproduced with permission [108]. Copyright 2019, Elsevier Ltd. (c) Schematic diagram of the flexible piezoelectric transistor. (d) Performance test of the flexible piezoelectric transistor: images and results of quantitative measurement of the bend angle of a human wrist (the bending angles are 5°, 30°, and 60°). Reproduced with permission [57]. Copyright 2020, Elsevier Ltd.

Yin et al. [108] proposed a photocrosslinkable elastic ionic polyacrylamide hydrogel (EIPH) to fabricate high-sensitivity low-energy consumption capacitive and organic thin-film transistor (OTFT) pressure sensors. The EIPH was prepared with a solution process by photopolymerization of an acrylamide monomer in an aqueous solution of poly (acrylic acid) and CaCl_2_. It was then micropatterned on an indium-tin oxide electrode. Figure 14(a) schematically shows the operation mechanism of the micropatterned EIPH-based capacitive pressure sensor. When the pressure is applied on the sensor, the micropillar EIPH structures deform, and the contact area between the electrodes increase, resulting in the increased charge accumulation at the interface between the electrode and the hydrogel. Therefore, capacitance of the sensor will increase with the applied pressure, as shown in Figure 14(b). 10-μm-wide EIPH micropillar structures achieved a sensitivity of 103.8 nF/kPa in the pressure range of 0–3 kPa. The high capacitance sensitivity is due to the formation of an electrical double layer. Furthermore, a proof-of-concept capacitive matrix-type pressure sensor was prepared by laminating the micropatterned EIPH coated ITO/PET substrate with a 4 × 4 electrode array. The array can be used for tactile sensing to demonstrate pressure mappings with ‘O’ and ‘K’ shapes. The sensor array shows great potential for multitouch devices. Such EIPH-based OTFT pressure sensors not only have high sensitivity, but also have low operation voltage, which have broad application prospects in wearable devices.

#### 3.2.3. Artificial tactile system based on piezoelectric-type tactile sensor

Piezoelectric materials can convert mechanical pressure into electrical signals directly and efficiently. And piezoelectric tactile sensors possess the priorities with low power consumption and high sensitivity. It would play an important role in artificial intelligence, advanced manufacturing, and smart wearable devices. However, it should be noted that it is difficult to capture electric signal triggered with small deformation of the piezoelectric materials under a weak force. Thus, with signal amplification activities of FETs, integration of piezoelectric pressure sensors with FET would be interesting. It would have wide potentials in electronic skin field and brain-inspired perceptual cognitive system.

Wang et al. [57] constructed a high-performance, energy-efficient, and fully flexible piezoelectric tactile sensor based on piezoelectric effect, as shown in Figure 14(c). It combines piezoelectric material with mechanical-to-electrical conversion function of β polyvinylidene fluoride (PVDF) nanorod arrays and organic field-effect transistor (OFET) devices with signal amplification function. Here, the gate voltage of FET is provided by the piezopotential generated by the mechanical response of piezoelectric materials. The nanorod arrays considerably improve the piezoelectric properties of PVDF. The piezoelectric electronic pressure sensor perfectly combines the piezoelectric effect and FET devices. Most importantly, this piezoelectric tactile sensor can not only quantitatively identify different masses, but also real-time detect the curvature of human wrist (0-90°) (Figure 14(d)). Under bending at a certain angle, the output curve exhibited similar symmetry, evidencing the reliability. Moreover, the current is highly dependent on the bending angles.

#### 3.2.4. Artificial tactile system based on triboelectric-type tactile sensors

**Figure 15.**
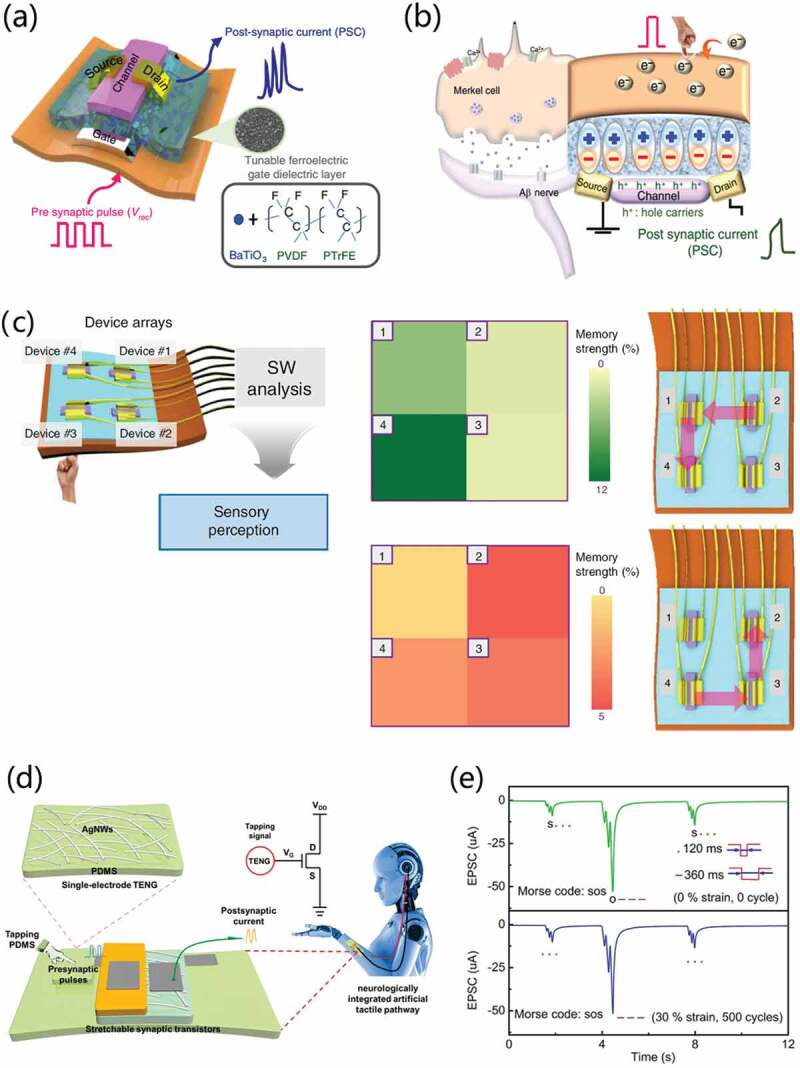
(a) Intrinsic-synaptic tactile sensory organ (AiS-TSO) structure diagram. (b) Sketch of device structure corresponding to a Merkel cell and Merkel cell-neurite complexes (MCNCs) structure. (c) Schematic illustrations of the AiS-TSO showing reception and preprocessing in parallel and the intelligence. Reproduced with permission [87]. Copyright 2020, springer nature. (d) Schematic illustration of a self-powered integrated tactile pathway with mechanoreceptor and synapse. The inset showing the circuit diagram of the integrated tactile pathway. (e) Tactile single-triggered EPSC amplitudes of the stretchable vertical synapse with Morse code of ‘SOS’. Reproduced with permission [110]. Copyright 2021, Elsevier Ltd.

Merkel cells are cutaneous mechanosensitive cells that form synapses with afferent neurons. These complexes are referred to as Merkel cell-neurite complexes (MCNCs) [109]. Inspired by the structure and intelligent functions of Merkel cell-neurite complexes, Lee et al. [87] reported a flexible, intrinsic-synaptic tactile sensory organ (AiS-TSO) that could mimic synapse-like connections using an organic synaptic transistor. Barium titanate nanoparticles and poly(vinylidene fluoride-trifluoroethylene) nanocomposite act as ferroelectric gate dielectric, as schematically shown in Figure 15(a). Figure 15(b) shows the device structure corresponding to a Merkel cell and MCNCs structure. Touch stimulation induces alignment of dipoles within the ferroelectric gate dielectric by triboelectric-capacitive coupling effect that causes the modulation of post-synaptic current signal, thereby allowing tactile information to be imparted in a self-energy transducer manner. Moreover, through multiple functions of slowly adapting, filtering and memory, the synaptic function enables the output signal to be pre-processed. Besides, it was demonstrated that the AiS-TSO device has adaptation, filtering, and memory functions and shows parallel spatiotemporal reception and preprocessing of tactile information (Figure 15(c)). With the modulation of composition of the nanocomposite ferroelectric layer, it is also possible to tune the synaptic weight of the Ais-TSO. Furthermore, a 2 × 2 sensor array is proposed with the function of recognizing the number and order of touch without additional signal processing after all stimuli ceased. Figure 13(c) shows the reception and preprocessing with the expected order (2→1→4→ and 4→3→2).

In somatosensory systems, the integration and cooperation of receptors and synapses enable human body to effectively sense and process information. Wang et al. [110] developed a stretchable self-powered neurologically integrated artificial tactile platform. It couples tactile-sensitive triboelectric nanogenerator (TENG) and invented stretchable vertical organic field effect transistor (Figure 15(d)). The transistor demonstrates low working voltage of ~0.5 V, high current density of 5.19 mA/cm^2^ and excellent stretching stability. TENG converts the tactile information into electrical signals and the stretchable vertical synaptic transistor plays the function of signal conversion and neuromorphic operations to emulate the biological functions. It is worth noting that the integrated artificial tactile pathway has good mechanical stability and biological synaptic simulation function under different tensile tests. In addition, the international Morse code (showing the ‘SOS’ Morse code, which is the most common distress signal) and pressure ulcer of human skin have been successfully mimicked on the integrated artificial tactile pathway, as shown in Figure 15(e). At both 0 stretching cycle with 0% strain and 500 stretching cycles with 30% strains, emergency signal ‘SOS’ can be recognized. These results indicate that stretchable vertical organic field effect transistors have great potentials in wireless communication, bio-inspired neuromorphic systems, future intelligent robotics and human-machine interfacing.

Human beings use different types of skin receptors to detect tactile stimuli by combining pressure and vibration signals. Various tactile sensors based on different conduction mechanisms have been explored to construct artificial tactile sensing systems with perception and memory functions. These tactile sensors include resistive-type, capacitive-type, piezoelectric-type and triboelectric-type [60,111]. Resistive-type tactile sensor has the advantages of high sensitivity, simple device structure and fabrication process. It is a general method to construct artificial tactile sensing system by coupling resistive-type tactile sensor with neuromorphic devices. In addition, tactile sensor based on triboelectric effect has attracted extensive attention due to its simple structure, high efficiency and energy-saving characteristics. In a word, different technical solutions could be selected in different application scenarios, as well as a wide selection of material systems. At the same time, the artificial tactile sensing system based on tactile sensors and neuromorphic devices has realized various advanced biological perception and learning recognition functions. As has given new vitality to intelligent robots and human-computer interface.

Though the studies of artificial tactile sensing system has made great progress, there are still great challenges to achieve various tactile sensing functions similar to that of human skin. On the one hand, the existing artificial tactile sensing system is still at the level of limited number of devices. Moreover, it is difficult to realize parallel signal processing in large area [112]. Therefore, realization of highly arrayed sensors and neuromorphic devices coupled together to build artificial tactile sensing systems is an important research branch in the future. On the other hand, to construct a complete artificial tactile sensing system, it is necessary to imitate multiple sensory systems. In addition to physical pressure sensors, there a limited reports on strain and temperature sensors in artificial tactile sensing. Thus, it is inevitable to develop a multifunctional collaborative artificial tactile sensing system that can not only perceive physical pressure and external temperature, but also realize bending and stretching in the future.

### 3.3. Artificial auditory perception system

Auditory system is one of the most important perceptual systems that can detect, process and store acoustic signal, making human information exchange more efficient and direct. The auditory organ is a functional unit to feel the sound wave in human body. With the help of auditory organ, we can obtain various kinds of sound information, so as to communicate with each other, avoiding enemies, capturing prey, and so on, which is of great significances to our daily life activities [113]. The generation of human auditory system is a complex biological process. Auditory receptors are the Corti’s organ in the basilar membrane of the cochlea [114]. When external object vibrates to produce sound, periodic changes in air pressure in the sound wave cause the eardrums to vibrate with accurate frequency and amplitude, correspondingly. These mechanical vibrations can be transmitted to the cochlear hair cells through the auditory bone and can be converted into electrical signals to cause auditory nerve impulses. Finally, these signals enter the auditory area of the cerebral cortex, resulting in human auditory sense. Furthermore, accurate sound location in nervous system of animals plays an important role in information exchange and intelligent activities. In our human neural system, sound location function is achieved by detecting the time differences between sound signals received by two ears. On the one hand, vibration is one of the most powerful communication modes for information sharing and azimuth state recognition. Therefore, patients with auditory system failure have serious auditory perception and verbal communication obstacles. In order to provide alternative treatment options, researchers in multiple interdisciplinary areas have been trying to develop low-cost, low-power, convenient and stable artificial auditory systems to help people with auditory obstacles to restore or improve their auditory perception ability [115,116]. On the other hand, flexible and stable artificial auditory system will give next generation of intelligent robots new vitality. With the artificial auditory system, the intelligent robots would have the ability to locate and track sounds. As would endow the robots decision-making ability based on hearing, speaking and understanding.

**Figure 16.**
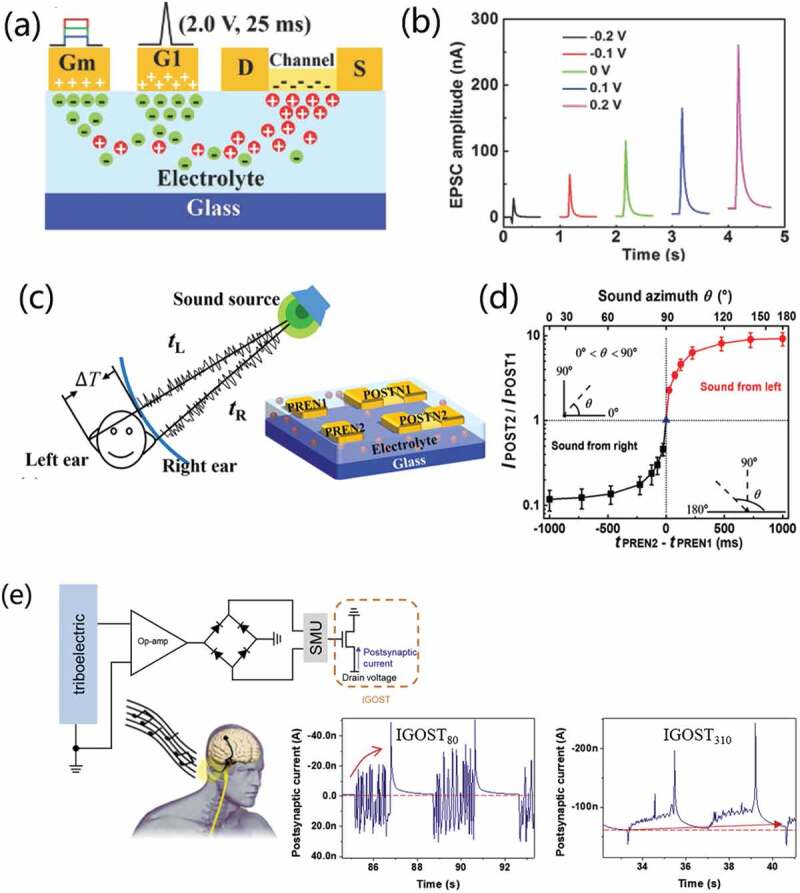
(a) Schematic structure of multiterminal neuro-transistor with one input gate (G1) and a modulatory gate (Gm). (b) EPSCs triggered by an electrical pulse (2.0 V, 25 ms) applied on G1 with different V_Gm._ (c) Schematic image of sound location by binaural effect in the human brain. (d) Sound azimuth detection emulated on the multiterminal neuro-transistors. Reproduced with permission [21]. Copyright 2019, WILEY-VCH Verlag GmbH. (e) a circuit diagram of an auditory nerve system and schematics of human auditory nerve systems. Reproduced with permission [80]. Copyright 2019, Elsevier Ltd.

Figure 16(a) depicts a capacitively lateral coupled multi-terminal oxide-based neuron transistors [21]. It uses solution processed chitosan-based polysaccharide electrolyte as gate dielectric and indium gallium zinc oxide as semiconductor channel. In addition, a modulatory terminal is designed for the device to modulate biological synaptic behaviors, which greatly enriches the regulatory methods of synaptic plasticity. As shown in Figure 16(b), EPSC amplitude triggered by the same presynaptic spike (2.0 V, 25 ms) applied on G1 increases with the increased bias applied on modulatory terminal (G_m_). The results indicate that the synaptic weight can be modulated by the modulatory gate. More interestingly, the dendritic discriminability of different spatiotemporal input sequences is successfully simulated by applying spatiotemporal electrical pulse sequences on the lateral gate electrodes on the multi-terminal oxide neuron transistor. As a powerful proof of spatiotemporal information processing, a simple artificial neural network based on this multi-terminal oxide-based neuron transistor is constructed to simulate the sound azimuth detection function of the human brain. As shown in Figure 16(c), the system is composed of two lateral gates and two pairs of source/drain terminals that can be regarded as PRENs (PREN1 and PREN2) and POSTNs (POSTN1 and POSTN2). While PREN1 and PREN2 can be regarded as left ear and right ear sound sensing neurons, respectively. Electrical pulses (2.0 V, 25 ms) were applied on PREN1 and PREN2, mimicking the sound received on the ears. The time interval between the two stimuli indicates the interaural time difference. Thus, ratio between the two EPSCs hints the sound azimuth, as shown in Figure 16(d). Sound azimuth detection function could also be mimicked using resistive switching synapses. Wang et al. [117] proposed a spatiotemporal neural network with resistive switching synapses. The time-coded spikes are reshaped into exponentially decaying signals, and the time difference of spikes among different neurons provides spatiotemporal coding with high sparsity and high information capacity. Recognition of spike sequences is demonstrated after supervised training of a multiple-neuron network with resistive switching synapses. Thanks to the high sensitivity to spike timing, sound azimuth detection function is mimicked.

Seo et al. [80] developed an ion-gel gated organic synaptic transistor (IGOST) using poly(thienoisoindigo-naphthalene) (PTIIG-Np) film as channel layer and 1-ethyl-3-methylimidazolium bis(trifluoromethylsulfonyl)imide ([EMIM][TFSI]) based ion-gel as gate dielectric. The synaptic decay constant of the synaptic transistors can be tailored with crystallinity of the PTIIG-Np semiconductor without changing the form of presynaptic spikes, which enables broad application potentials from neuromorphic computing to neuro-prosthetics. The crystallinity controlled the electrochemical-doping kinetics and the resultant synaptic behaviors. Thus, the device demonstrated not only long-term retention for learning and memory but also the short-term retention for fast synaptic transmission. As is useful for emulating peripheral nerves such as sensory and motor nerve. Furthermore, an artificial auditory system was constructed by coupling IGOST with acoustic sensors (triboelectric sensors), as schematically illustrated in Figure 16(e). Rectified output voltage pulses from acoustic sensors were transmitted to the gate electrodes of IGOST. In this auditory system, a speaker generated a 5 Hz square acoustic wave. The acoustic sensor converts it into electrical signal, and the amplified and rectified output voltage pulse is applied to the gate of IGOST. For IGOST_80_, the 5 Hz sound wave induced EPSC potentiation that decayed rapidly when the sound ends. In contrast, for IGOST_310_, the sound wave induced LTP and slow synaptic decay. Thus, after repeated sound detection, resting current increases. Here, the modulation of IGOST operation mode allows for versatile neuromorphic electronics.

**Figure 17.**
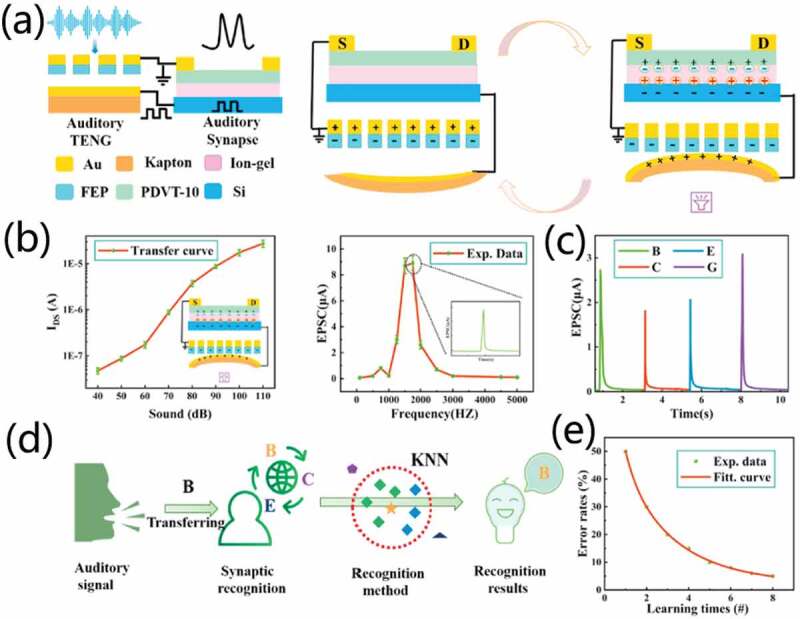
(a) Schematic diagram of artificial auditory pathway and the working principle of TENG actuated artificial auditory pathway. (b) Sound pressure level and frequency dependent performances. (c) EPSC under different word instructions (B, C, E, G) with sound level of 90 dB. (d) Illustration of the acoustic signal recognition process. (e) Recognition error rates of ‘B’ after 8 times training. Reproduced with permission [51]. Copyright 2020, Elsevier Ltd.

Liu et al. [51] developed a self-powered artificial auditory pathway consisting of a triboelectric nanogenerator (TENG) and a field-effect synaptic transistor (FEST), as schematically shown in Figure 17(a). The triboelectric nanogenerator (TENG) and field-effect synaptic transistor (FEST) were deemed as acoustic receptor and acoustic synapse, respectively. TENG converts voice information into electrical signals, while synaptic transistor processed the signal conversion and neuromorphic operations to emulate biological acoustic functions. The artificial auditory pathway demonstrates sound pressure level and frequency-dependent performances, as shown in Figure 17(b). The system demonstrates a promising performance with high sensitivity (129 mV/dB) in a wide frequency range from 50 to 5K Hz, which enables efficient and precise human-computer interaction. Figure 17(c) shows EPSC under different word instructions (B, C, E, G) with sound level of 90 dB. Each EPSC peak provides a special signal to match corresponding word, indicating the effectiveness of distinguishing letters. The system can also realize supervised learning by using k-nearest neighbors as classifier, as shown in Figure 17(d). Figure 17(e) shows the recognition error rate of word ‘B’, indicating an overall accuracy of 95% for the 7-word instructions after the 8th training. Sound azimuth detection can also be well achieved by constructing a neural network composed of two artificial auditory pathways. Two TENG acoustic receptors were regarded as left and right ear, and two synaptic transistors were regarded as FEST1 and FEST2, respectively. Sound source azimuth dependent post-synaptic current ratio of FEST1 and FEST2 were observed. Finally, a self-adaptation artificial auditory neuromorphic circuit with noise-adjustable behavior is demonstrated, which can well simulate the biological auditory pathway and provide the possibility for the practical application of artificial auditory pathway in complex environment.

Inspired by human auditory system, various materials and exquisite structures have been adopted for artificial auditory system applications. In addition, various non-traditional manufacturing methods have also been widely used in the construction of artificial auditory system, including basilar membrane-inspired flexible electronics, 3D printed hydrogel ear and magnetically charged haircells, and so on [118,119]. However, compared with artificial vision and artificial tactile system, the research work on artificial auditory system is still in its infancy, especially the artificial auditory system based on neuromorphic transistor is rarely reported. At present, artificial auditory system based on three-terminal neuromorphic transistors often constructs a simple neural network at the level of single device, conceptually realizing brain-like sound localization function. It is worth noting that the biological auditory system is a complex system. Currently, artificial auditory system based on neuromorphic transistors can only perceive environmental acoustic signals, and has great deficiencies in integrating, analyzing and storing massive sound information. Therefore, developments of artificial auditory system which can integrate perception, memory and analysis functions is an important research branch in the future. Moreover, compared with human auditory system, the development of artificial auditory system with intelligent adaptive and low power consumption is also essential.

### 3.4. Artificial olfactory perception system

Olfactory system is one of the basic sensory systems in human beings. When the human olfactory receptor is stimulated by gas in the environment, it produces nerve impulses, which will be transmitted into the nerve in the cerebral cortex. Then, human body will have olfactory sensation. Olfactory receptors, brain functional areas and muscles can coordinate with each other. Therefore, human body can quickly respond to dangerous complex environments [120,121]. Inspired by the biological olfactory system, researchers have proposed bionic olfactory receptors for detecting various odors in recent years. Transistor type gas sensor is one of the most promising gas sensors because of its small size, easy integration and multi-functions. Besides, functional nanomaterials can also be integrated with functional biomaterials to improve the sensitivity of the sensors in transistor configuration because of the signal magnification. These sensors have been widely adopted in various fields, including biomedical engineering, environmental monitoring, food safety and counter-terrorism, and so on [122]. Furthermore, researchers developed artificial olfactory perception systems by integrating gas sensors with synaptic devices to establish sensing, storage and computing platform, which realized advanced olfactory neuromorphic functions. These achievements would be of great significances for construction of neuromorphic computing systems and intelligent robots in the future.

**Figure 18.**
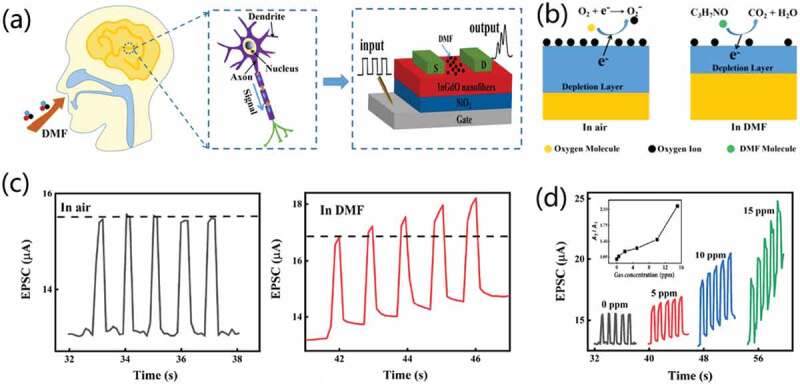
(a) Schematic diagram of signal transmission by the olfactory synaptic transistor after inhalation of DMF gas. (b) Reaction of DMF gas molecules on the surface of the InGdo nanofiber. (c) Responses of impulse stimulation in air environment and in DMF gas environment. (d) Responses to the impulse signal at different concentrations. Reproduced with permission [85]. Copyright 2022, American chemical society.

Li et al. [85] successfully prepared an olfactory synapses of InGdO nanofiber field-effect transistors for target gas detection, as shown in Figure 18(a). The olfactory synapse integrates human olfactory nerve sensing mechanism with the reduction reaction of dimethylformamide (DMF) with oxygen. Figure 18(b) shows the working mechanism of InGdO nanofiber field-effect olfactory synaptic transistors. It can induce and activate synaptic properties by adjusting oxygen vacancies in the active layer. In addition, the detection of target gas by olfactory synapse was simulated. As shown in Figure 18(c), there are no changes in the response amplitude under pulsed voltage stimulation with air ambient. While in DMF gas ambient, increased EPSC values were observed under pulsed voltage stimulation, suggesting an olfactory synaptic response to the DMF gas. Furthermore, with sequential gate stimuli with pulse width of 500 ms, frequency of 1 Hz, and amplitude of 1 V, the EPSC gain A5/A1 increases with the increased concentration of DMF, which is consistent with the responses of the olfactory organs in human body, as shown in Figure 18(d). Additionally, Pavlovian conditioned reflex experiment was also successfully simulated by testing the response under different concentrations of DMF pulses, when the synaptic transistor has associated the pulse signal with DMF gas. The proposed gas sensing device with learning and memory functions provides new ideas for the developments of high-performance gas sensors and bionic electronic devices.

In daily life and industrial environment, the detection of nerve agents is extremely important for safety. Yoo et al. [123] reported a bionic human olfactory receptor (hOR)-based on single-walled carbon nanotube-field effect transistor (SWCNT-FET) for the detection of dimethyl methylphosphonate (DMMP), a structural simulant of sarin and a chemical warfare agent (CWA). HOR2T7-conjugated bioelectronic nose (hOR2T7 B-nose) was proposed to selectively and sensitively detect the DMMP. Thus, SWCNT-FET can convert biological signals of hORs into electrical signals with high sensitivity. The results showed that DMMP with a concentration of 10 fM can be detected in hOR2T7B-nose. The target molecule was distinguished from an odor molecule with smoky smell. The results imply that the B-nose technology can be used for early response to nerve gas attacks in various situations, including terrorism and military threats, and so on. Besides, most of the existing transistor-based gas sensors use unipolar transistors, which usually have only one single response to gas detection and cannot detect multiple gases. Ambipolar transistors provide possibilities for highly selective sensing because of two types of charge carriers involved in the operation. Zhou et al. [59] demonstrated a high-performance gas sensor based on an ambipolar OFET fabricated with a 2,5-bis(4-biphenylyl) bithiophene (BP2T) and copper hexadecafluorophthalocyanine (F_16_CuPc) bilayer. The ambipolar transistor possesses great advantages of multi-parameter, high output signal and better stability compared to unipolar transistors. It demonstrates clear dual responses based on current signal to several gases, from oxidative to reductive gases, including NO_2_, NH_3_, H_2_S and SO_2_, exhibiting highly selective and sensitive gas detection.

High-performance transistor-based gas sensor arrays have also been proposed to assess complex gas environments. Yuan et al. [124] proposed an integrated array composed of multiple silicon-based, chemical-sensitive field effect transistors (CSFET) for food safety and quality detection. CSFETs were decorated with different functional sensing materials to be selective to different target gases. In other words, Ru and Ag are selected as sensing materials for the detection of ammonia (NH_3_), hydrogen sulfide (H_2_S). The CSFET sensors demonstrate good stabilities under all testing concentrations. Standard deviations in the responses towards 100 ppb pulses of NH_3_, H_2_S, and 80% RH are 5.2%, 1.5%, and 1.6%, respectively. A portable and autonomous sensor system is constructed by integrating the developed CSFET sensor array and printed circuit board (PCB). The multi-CSFET sensor array was tested with various levels of NH_3_, H_2_S, and humidity to characterize target and cross-sensitivities of each sensor in the array. The system exhibits stable room-temperature responses to NH_3_, H_2_S, and humidity and exhibits good selectivities in trace-level gas sensing. Kwon et al. [125] proposed an artificial olfactory system based on a spiking neural network (SNN) and FET-type gas sensors for quickly and reliably detecting toxic gases. The FET-type gas sensors with an In_2_O_3_ film as sensing material were fabricated to detect NO_2_ and H_2_S gases. A micro-heater is also integrated. Based on the gas sensing datasets, an SNN consisting of bio-plausible neurons and electrical synaptic devices can predict gas types and their concentrations. An ANN using backpropagation algorithm was applied to train the datasets. Then, the weights trained in the ANNs were transferred into synaptic weights in hardware-based SNN. The SNN using only 12 sensors demonstrate a low error rate in the order of ~ 3% in predicting the concentrations of NO_2_ and H_2_S. The results show that the hardware-based SNN is superior to the traditional machine learning algorithms. The results indicate that the artificial olfactory system based on hardware SNN and FET gas sensors has excellent performances in the detection of toxic gases.

**Figure 19.**
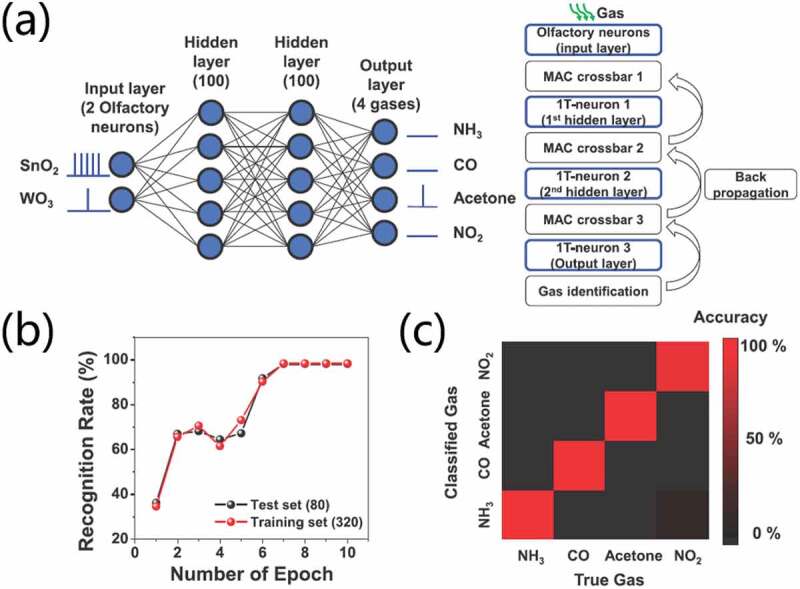
(a) Illustration of multi-layer spiking neural network (SNN) constructed for gas classification and the flow chart of the simulation. (b) Recognition rate as a function of the number of epochs for test set and training set. (c) the accuracy of test results. Reproduced with permission [52]. Copyright 2022, WILEY-VCH Verlag GmbH.

Integrating of sensing detection and information storage in electronic devices is of great significances for intelligent robots and medical health. Song et al. [84] proposed an effective and facile memory system for simulating memory impairments caused by exposure to hazardous gas (NO_2_). Due to the good gas sensitivity of organic semiconductors at room temperature, the artificial memory system can realize the integration of sensing detection and information storage just based on an OFET. The device exhibits desirable gas-controlled paired-pulse facilitation (PPF) memory behavior and can accumulate sensing signals for hazardous gas leakage. The accumulation of sensing signal triggered with single prolonged NO_2_ exposure and long-term repeated NO_2_ exposure allows the system to simulate the inhalation, metabolism and cumulative organ damage of human body upon hazardous gas pollution. Based on the above characteristics, the device has the bionic ability to simulate the damage of harmful gases to human organs. Han et al. [52] proposed an artificial olfactory neuron module for neuromorphic electronic nose (E-nose) application by hybridizing a chemoresistive gas sensor with a single transistor neuron (1T-neuron). Resistive gas sensors and single transistor-based neuron are made of semiconductor metal oxide (SMO) and metal-oxide-semiconductor field-effect transistor (MOSFET), respectively. 1T-neuron was fabricated with a MOSFET structure on a silicon on insulator (SOI) substrate. SnO_2_ and WO_3_ were adopted as sensing materials for gas sensors and were deposited on microheater platform in the form of nanocolumnar films using glancing angle deposition via RF sputtering. Au nanoparticles were also coated on SMO for catalytic effect. The working principle of the SMO gas sensor is related to the resistance modulation of the sensing material. The electron-depleted region on the surface of SMO would be modulated when gas is adsorbed onto the surface of SMO, which results in the changes of resistances of the SMO. The proposed E-nose can perform gas detection and spike coding at low power consumption. It simultaneously detects gas and encodes spike signals for sensor neuromorphic functioning. Semiempirical simulations with Python software were performed to classify four gases (NH_3_, CO, acetone, NO_2_). As shown in Figure 19(a), a four-layer SNN was constructed. Two input neurons corresponded to two artificial olfactory neuron modules with a SnO_2_ gas sensor and a WO_3_ gas sensor. While the four output neurons corresponded to four gas species. Training was performed with 320 training datasets and validation was performed with 80 test datasets. After seven epochs, gas classification with a high accuracy of 98.25% was addressed, as shown in Figure 19(b). More interestingly, neuromorphic electronic noses can classify different types of wines, as shown in Figure 19(c). The results prove that the artificial olfactory neuron module can act as an electronic sommelier.

In current researches, various materials have been used to prepare high-performance transistor-based gas sensors, including graphene, carbon nanotubes (CNT), In_2_O_3_ film, amorphous indium gallium zinc oxide and 2D materials (MoS_2_), and so on. Furthermore, artificial olfactory perception system is constructed based on various mechanisms, including artificial network algorithm for odor pattern recognition, artificial electronic nose constructed by integrating synaptic memory and gas detection function of neuromorphic transistors [125,126]. Unfortunately, most of the current transistor-based gas sensors just convert gas concentrations into electrical signals. They do not have the olfactory electronic nose function of sensing detection and information storage. Therefore, it would be one of the future research focus to construct high-performance artificial olfactory systems by coupling neuromorphic transistors with other electronic components.

### 3.5. Artificial gustatory perception system

Taste perception also provides abundant information about food and environments, that is, the five basic flavors (sweet, sour, bitter, salty and delicious). Taste receptors in the mouth are stimulated by the taste substances. Then, they transmit signals to the taste center of the brain through a neural sensory system that collects and transmits information. Finally, taste is generated through analysis of the comprehensive neural system of the brain. In addition, our tongue has other taste manifestations, such as astringency. Over the past decades, researchers have been trying to develop artificial taste sensors to identify five basic tastes. Inspired by human taste, artificial taste sensors have been successfully developed as electronic tongues. It promotes the detection of flavor agents in the field of industrial life and gives intelligent robots new vitality in perceptual learning.

**Figure 20.**
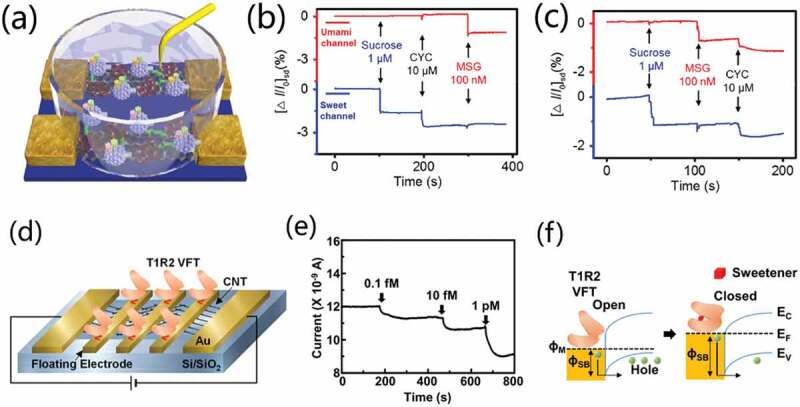
(a) Schematic diagram of a DBT using graphene FETs. (b) Real-time responses of two channels in the DBT sensor toward umami and sweet tastants. (c) Enhancing property for DBT. Reproduced with permission [53]. Copyright 2016, American chemical society. (d) Schematic diagram of bioelectronic tongue based on T1R2 VFT for sweet substances detection. (e) Real-time response by adding sucrose solution based on T1R2 VFT. (f) Sensing mechanism of the device. Reproduced with permission [76]. Copyright 2022, American chemical society.

Ahn et al. [53] proposed a duplex bioelectronic tongue (DBT) using graphene FETs, as schematically shown in Figure 20(a). Micropatterned graphene surfaces were functionalized with two types of nanovesicles, that is, human T1R1/T1R3 for the umami taste and human T1R2/T1R3 for the sweet taste. Based on graphene transistor sensors with high stability and fast response characteristics as well as high sensitivity originated from selective interaction of each channel, duplex bioelectronic tongue (DBT) can detect umami and sweet tastants simultaneously. Figure 20(b) shows real-time responses of two channels in the DBT sensor toward umami and sweet tastants. The DBT platform exhibits high sensitive and selective recognition of target tastants at low concentrations (ca. 100 nM). In addition, the developed DBT can detect the enhancing effect of taste enhancers as in a human taste sensory system, as shown in Figure 20(c). The technique would be a useful tool for detection of tastes instead of sensory evaluation and development of new artificial tastants. Besides, Capitán et al. [127] used electronic tongue for sensory testing and prediction of drinking water from different origins. The electronic tongue array consists of six ion-sensitive field-effect transistors (ISFET)-based sensors, one conductivity sensor, one redox potential sensor, two amperometric electrodes, one gold microelectrode and one nanocomposite planar electrode. Interestingly, the proposed electronic tongue obtains a good classification according to their chemical composition, including hardness, alkalinity, chlorine content, and ionic content. The results showed that the electronic tongue had the ability to analyze the sensory characteristics of water samples, and had considerable prospects in replacing the taste panel.

Jeong et al. [77] proposed an ultrasensitive taste bioelectronic tongue device using carbon nanotube field-effect transistor (CNT-FET) functioned with human sweet receptor T1R2 Venus flytrap (VFT). Figure 20(d) schematically shows a T1R2 VFT-based bioelectronic tongue for the detection of sweet substances. The T1R2 VFT was immobilized on the gold floating electrodes of the CNT-FET using a cysteine linker. Here, the T1R2 VFT receptor was used as function unit in the artificial sweet sensing system, immobilized on floating electrodes of a CNT-FET. Figure 20(e) shows real-time responses of the T1R2 VFT-immobilized taste bioelectronic tongue to various concentrations of sucrose. It is worth noting that the bioelectronic tongue can detect sweetener solution with concentration as low as 0.1 fM. The high sensitivity can be attributed to small size of T1R2 VFT receptor. Figure 20(f) shows the operation mechanism. When T1R2 VFT bounds the target sweet substance, work function of the floating electrode (ϕ_M_) will decrease. Thus, Schottky barrier height (ϕ_SB_) increases, which induces the decrease in the hole-carrier currents. Interestingly, the device also selectively distinguish sweet substances from other taste substances, just like what human tongue does. Furthermore, the bioelectronic tongue exhibits high stability during long storage and can be used repeatedly. The proposed device can detect responses to sweet substances in real food environments such as apple juice and chamomile herb tea. Based on these results, the developed sweet bioelectronic tongue would have great application potential in food industry and basic research.

As the main representative artificial taste system, bioelectronic tongue has aroused wide interests of researchers. However, the gustatory sensors need to integrate biological receptors into devices to detect target flavor. Therefore, it is necessary to consider the biocompatibility with functional materials of the devices, cell-device interaction and other forms of biological contamination during the device processing and cell seeding. All these increase the difficulties in the improvements and progresses of artificial electronic tongue devices to some extent [128]. Presently, studies of artificial taste sensing system based on three-terminal neuromorphic transistor are still in its infancy. It is mainly focused on the detection of specific taste at the level of a single device level combined with human taste receptors. In addition, there are some reports on the construction of electronic tongue devices using other electronic components and transistors. But, the function is also limited to the detection of target flavor agents on the basis of specific taste receptors. As comparison, human taste system is a very complex and powerful perception system. It can not only sense and distinguish the basic sweet, sour, bitter, salty and delicious, but also is very sensitive to temperature perception. Thus, there is still a long way to realize similar function based on neuromorphic transistors as what human taste system has. Therefore, it would be a possible direction for the future research to seek new functional materials and develop fast response, high sensitivity and selectivity neuromorphic devices by micro-nano fabrication. Naturally, development of multifunctional taste sensors with combination and synergy in complex flavor reactions is of great importance in the near future.

### 3.6. Artificial multi-modal perception fusion system

When interacting with the real, dynamic world, our activities are extremely complex. Each perception system always provides rich surrounding information to our brain to make decision. Thanks to the innovation of various functional materials and device structures, there are rapid progresses in neuromorphic devices to shape and imitate human perception systems. As mentioned previously in this review, artificial visual system, artificial tactile system, artificial auditory system, artificial olfactory system and artificial taste system have been constructed based on three-terminal neuromorphic transistors. Several functions of corresponding sensory systems have been imitated [47,129]. It is worth noting that human perception system is a complex system with multi-cue analysis, good integrity and synergy. However, there are few reports on artificial neuromorphic systems with multi-perception fusion. Therefore, the developments of neuromorphic platforms with multi-perceptual fusions will provide great significances to artificial intelligence and biomedicine.

**Figure 21.**
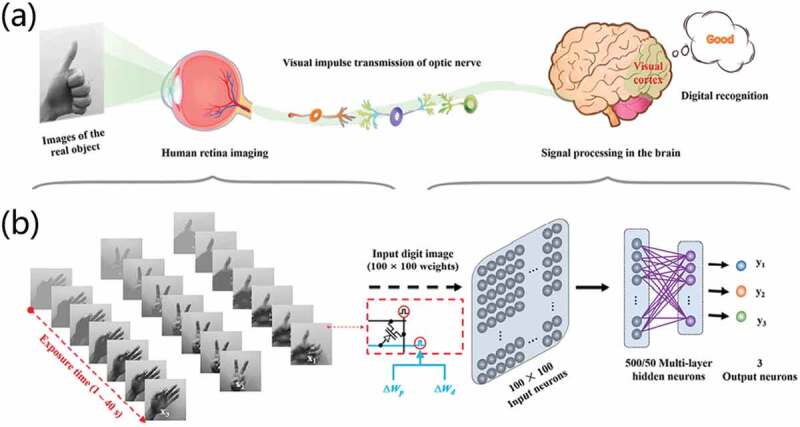
(a) Schematic diagram of brain image recognition process. (b) Neuron network structure for gesture pattern recognition. Reproduced with permission [47]. Copyright 2022, American chemical society.

Liu et al. [47] reported a nanowire-channel intrinsically stretchable neuromorphic transistor (NISNT). It uses electro-hydrodynamically printed P3HT/PEO NWs, possessing the ability to perceive tactile and visual information. It is commendable that NISNT is stretchable and can be closely connected to the fingers as a stretchable artificial nervous system for gesture recognition. The NISNT was attached to a finger. Then, the response was measured in a straight state and under bent to angles of 30° and 45°, respectively. When the bending angle increases, the device was stretched together with the deformation of the skin. Thus, the maximum EPSC triggered by 50 consecutive presynaptic spikes (−3.5 V, 50 ms) decreases. When NISNTs were attached to a knuckle in the thumb, index, middle, ring, and little finger, different gestures could be distinguished, including ‘Good’, ‘Yeah’, and ‘OK’ by comparing the strain current for pattern recognition. In addition, due to the light-sensitive feature of the P3HT/PEO channel, NISNT is used as a wearable artificial afferent nerve for light perception. Thus, it can sense the light stimulation from the external environment. Most importantly, NISNT combines visual information to further improve the accuracy of gesture recognition and achieve high-precision pattern recognition. Authors have simulated the responses of a 100 × 100 device array, which can sense light from the environment, transmit signals to the retina and input the signals via the optical nerve, as schematically shown in Figure 21(b). The pixel information was transformed to postsynaptic current and input into the artificial neural network. Backpropagation algorithm is used for training. The neural network can capture the features of the gestures and realize pattern recognition at high accuracy of ~96.3%. The work shows that the artificial neuromorphic system integrated with tactile perception and visual perception is of great significances to the construction of human-like perception system in the future.

**Figure 22.**
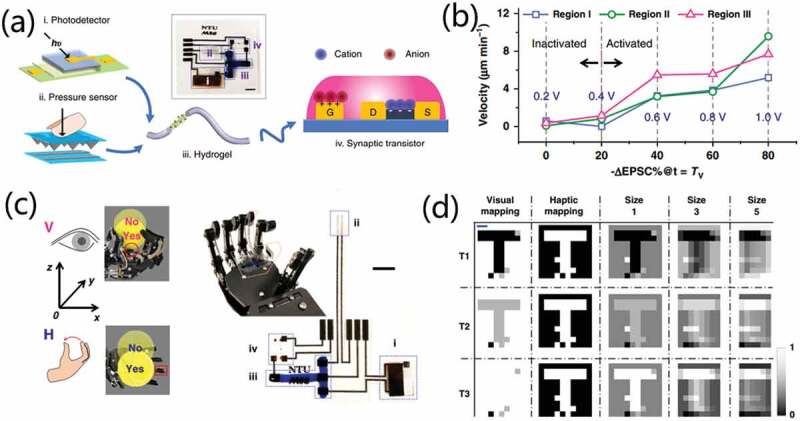
(a) Schematic diagram of BASE patch for visual-haptic fusion. (b) Response of the bimodal artificial sensory neuron to external stimuli. (c) the strategy of integrating visual and tactile feedback to realize the motion control of the robot. (d) the normalized mapping results based on unimodal information (visual or haptic) and VH fusion information. Reproduced with permission [48]. Copyright 2020, springer nature.

Inspired by the supermodal sensory fusion in sensory nervous system, Wan et al. [48] developed a bimodal artificial sensory neuron (BASE) based on ionic/electronic hybrid neuromorphic electronic devices to implement the visual-haptic fusion, as schematically shown in Figure 22(a). The BASE collects optical and pressure information from photodetector and pressure sensors, respectively. Then, the bimodal information can be transmitted through an ionic cable. At last, information is integrated and induces post-synaptic currents on a synaptic transistor. Figure 22(b) shows the responses of the bimodal artificial sensory neuron to external stimuli. In addition, based on bimodal information sensory cues, sensory neurons can be stimulated at multiple levels to successfully simulate motion control, manipulating skeletal myotubes and a robotic hand, as shown in Figure 22(c). More interestingly, by simulating the multi-transparency pattern recognition task, the enhanced recognition ability realized on the fusion of visual/haptic cues is confirmed, as shown in Figure 22(d). The results show that the highly integrated perceptual system constructed by simulating sensory fusion at the neuron-level has far-reaching significances for neurorobotics and artificial intelligence.

Zhang et al. [130] proposed a composite p-i-n junction synaptic transistor (p-i-n JST). In the transistor, n-type TiO_2_ film is covered with poly (methyl methacrylate) (PMMA) and with a p-type P3HT/PEO nanowire (NW) on top. The polarity of the charge carriers in the conductive channel can be modulated by altering the external inputs. Thus, the synaptic transistor can emulate the multiplexed neurotransmission of different neurotransmissions (i.e. glutamate and acetylcholine), so as to quickly switch plasticity between STP and LTP. The hole transport characteristics in the p-type P3HT NW is related to the formation of STP. While the electron transport characteristics in the n-type TiO_2_ layer and the trapping activities under the PMMA inversion layer are related to the formation of LTP. Interestingly, the responses of PMMA/TiO_2_ emulates the synergistic effect of taste and aroma on the control of food-intake behavior by using two parallel inputs on two gates. Thanks to the bipolarity, the p-i-n JST can also simulate distinct responses of gustatory receptor neurons to different concentrations of salt. Wan et al. [46] also proposed an artificial multimodal sensory-memory system with similar biological sensory memory ability. It is composed of sensors and flexible carbon nanotube synaptic transistors. The sensors can generate biomimetic visual, auditory, tactile inputs. While the CNT synaptic transistor possesses synapse-like signal processing and memorizing activities. By using ferroelectret nanogenerator (FENG) as tactile sensor, acoustic sensor and using phototransistor as optical sensor, three out of the five human basic sensing abilities can be realized, including touch, hearing, and sight. Based on the memory and learning characteristics of the sensory-memory system, the well-known psychological model describing human memory, that is, the ‘multistore memory’ model, and the classical conditioning experiment, i.e, ‘Pavlov’s dog’s experiment’, have been implemented electronically using actual physical input signals as the sources of the stimuli. The proposed artificial multimodal sensory-system can be used to simulate biological sensory and nervous system, which is conducive to the construction of environmental-interactive artificial intelligence.

Recently, various new materials endowed with new functions and new structures have been proposed for applications in neuromorphic devices and sensors. Thus, it is possible to develop artificial neuromorphic systems that can simulate human multi-modal perception functions. In addition, since our human perception system is a complex and multifunctional nervous system, it cannot be limited to developing a single-function artificial perception system at a single device level. Interestingly, with the help of new functional materials and diversified neuromorphic device structures, researchers have successfully realized visual-tactile fusion of artificial sensory neurons, complex neural behaviors generated by the synergistic effect of taste and olfactory sensory nerves, and multimodal artificial sensory-memory system with similar biological sensory-memory ability. Nevertheless, more works are still needed to achieve complete human perception activities, in terms of perception fusion system. Therefore, developing artificial multimodal sensory-systems with biocompatibility, high sensitivity and low power consumption will be an inevitable step in the developments of advanced intelligent human-computer interaction platform.

## 4. Summary and outlook

In recent years, various materials with functionalized structures have been designed for neuromorphic transistors, which deeply promotes the progresses of synaptic bionics and neuromorphic engineering. Besides, inspired by the powerful perception functions of human multi-sensory learning activities, developing artificial perception system is of great significances for artificial intelligence and humanoid robots. So far, neuromorphic devices have been proposed for applications in constructing artificial perception systems with complex sensing functions. The present review summarizes the device structure, working mechanism, biological synapse simulation and advanced neuromorphic applications of three terminal neuromorphic transistors. The review classifies and summarizes the reported artificial perception systems based on tri-terminal transistors, including artificial vision, tactile, auditory, olfactory and taste perception systems.

Up to date, tactile sensors based on different conduction mechanisms have been used to construct artificial tactile systems, including resistive-type, capacitive-type, piezoelectric-type and triboelectric-type tactile sensors. Moreover, studies on optoelectronic synaptic devices have also made progresses rapidly thanks to the excellent optoelectronic characteristics of advanced optoelectronic materials. Various types of artificial visual perception systems based on neuromorphic transistors have been reported. Unfortunately, studies of artificial auditory, olfactory and gustatory perception system based on neuromorphic transistors is still in its infancy. Though the construction of artificial perception system based on neuromorphic transistors has achieved fruitful results, there are still several challenges. First of all, the current artificial perception system based on transistors is generally constructed at the level of a single device. Therefore, it is still difficult to achieve the low power consumption, massively parallel processing, and processing in memory at system level. Furthermore, stability, durability and compatibility need to be improved in terms of neuromorphic transistors. It is worth noting that the construction of artificial perception system at the present stage mainly depends on the coupling between neuromorphic devices and other electronic components. Therefore, it is essential to develop neuromorphic devices with strong compatibility, high sensitivity and adjustable conductance. Finally, human perception is a complex and energy-efficient system. It is the basis of real-time dynamic interaction between human body and real world. At present, artificial perception mainly focuses on single function. Thus, it is highly desirable to develop a multi-sensor fusion system.

Benefiting from the properties of neuromorphic transistors, including operating mode of read-write separation, rich dynamic characteristics, good channel regulation ability and low power consumption, it provides more possibilities for realizing neuromorphic computing. In short, studies on artificial perception system based on neuromorphic devices is become a new branch of neuromorphic engineering. In the near future, constructing multimodal artificial perception system will become an indispensable link, which will inject new vitality into the development of artificial intelligence and the neuromorphic engineering.
